# Effect of small-sided team sport training and protein intake on muscle mass, physical function and markers of health in older untrained adults: A randomized trial

**DOI:** 10.1371/journal.pone.0186202

**Published:** 2017-10-10

**Authors:** Jacob Vorup, Mogens Theisen Pedersen, Lena Kirchner Brahe, Pia Sandfeld Melcher, Joachim Meno Alstrøm, Jens Bangsbo

**Affiliations:** Copenhagen Centre for Team Sport and Health, Department of Nutrition, Exercise and Sports, Section of Integrative Physiology, University of Copenhagen, Copenhagen O, Denmark; Universidad de las Palmas de Gran Canaria, SPAIN

## Abstract

The effect of small-sided team sport training and protein intake on muscle mass, physical function, and adaptations important for health in untrained older adults was examined. Forty-eight untrained older (72±6 (±standard deviation, SD) years men and women were divided into either a team sport group ingesting a drink high in protein (18 g) immediately and 3 h after each training session (TS-HP, n = 13), a team sport group ingesting an isocaloric drink with low protein content (3 g; TS-LP, n = 18), or a control group continuing their normal activities (CON, n = 17). The team sport training was performed as ~20 min of small-sided ball games twice a week over 12 weeks. After the intervention period, leg muscle mass was 0.6 kg higher (P = 0.047) in TS-HP, with no effect in TS-LP. In TS-HP, number of sit-to-stand repetitions increased (1.2±0.6, P = 0.054), time to perform 2.45 m up-and-go was lower (0.7±0.3 s, P = 0.03) and number of arm curl repetitions increased (3.5±1.2, P = 0.01), whereas in TS-LP only number of repetitions in sit-to-stand was higher (1.6±0.6, P = 0.01). In TS-LP, reductions were observed in total and abdominal fat mass (1.2±0.5 and 0.4±0.2 kg, P = 0.03 and P = 0.02, respectively), heart rate at rest (9±3 bpm, P = 0.002) and plasma C-reactive protein (1.8±0.8 mmol/L, P = 0.03), with no effects in TS-HP. Thus, team sport training improves functional capacity of untrained older adults and increases leg muscle mass only when ingesting proteins after training. Furthermore, team sport training followed by intake of drink with low protein content does lower fat mass, heart rate at rest and level of systemic inflammation.

Trial Registration: clinicaltrials.gov NCT03120143

## Introduction

A major challenge for older adults is to maintain physical function and health. Everyday activities, such as walking stairs and rising from a chair may be impaired with old age and affect independence as well as general well-being [1;2]. In addition, the risk of developing type 2 diabetes and cardiovascular diseases increases with old age [3;4]. Aging is also associated with a decline in muscle mass [5] and longitudinal studies have shown that from the age of 75 years the annual decline in muscle mass is about 0.7% and 0.9% in women and men, respectively [6]. Regular exercise can attenuate the loss in muscle mass and physical function associated with aging [7–9] and positively affects markers of metabolic function, i.e. blood lipids and insulin resistance [10;11].

Regular exercise organized as small-sided soccer games have shown to promote a broad range of beneficial adaptations important for physical function and health in young and old untrained adults [12]. Recently, regular small-sided floorball training has also been shown to be effective in lowering blood lipids as well as heart rate at rest and during submaximal exercise, in addition to improving physical function and muscle strength in recreational active men aged 69 (range 65–76) years with only a weekly training volume of ~50 min [13]. However, participants in the latter studies examining small-sided soccer and floorball training were highly active with ~8300 steps per day [13;14], and they had a higher than normal physical function exemplified by 16–19 sit-to-stand repetitions in 30 s vs. age-adjusted average value of ~15 repetitions [7;13]. Older untrained adults (up to 90 years) with lower physical capacity and with no experience with intense training may not endure regular team sport training, i.e. small-sided ball games, involving multiple periods of high intensity and several intense actions. Moreover, combining various small-sided ball games may lead to different physiological responses; e.g. soccer mainly involves multiple lower body activities, whereas floorball is also characterized by a number of upper body actions. Thus, examining training adaptations important for health as well as feasibility for training organized as various small-sided ball games in older (up to 90 years) untrained adults with a low physical capacity is important for the understanding of how exercise can affect health and physical function of the elderly.

Small-sided ball game training seems to have age-dependent anabolic effects on muscle mass. Thus, in young untrained adults, muscle mass has been shown to increase with two weekly 1-h sessions of small-sided soccer games for 12 weeks [15]. In contrast, in untrained older men aged 65–76 years no gain in muscle mass was observed neither after 4 and 12 months of regular soccer training [7], nor after 3 months of floorball training [13]. A lack of increase in muscle mass in older adults may be due to 'anabolic resistance', which is characterized by an impaired ability to increase muscle mass because of a limited ability to utilize protein after an anabolic stimulus, e.g. exercise [16]. It is possible that the total intake or the timing of the intake of protein in relation to training was insufficient to induce gains in muscle mass in the latter studies examining small-sided soccer and floorball. The diet recordings from Andersen et al. (2016) indicated that total daily protein intake was high (1.7 g/kg ~22% of daily energy intake). Thus, it may be that that the protein intake after training sessions was not sufficient, since it has been shown that the supply of amino acids immediately after exercise is important for promoting synthesis of muscle proteins [17]. Ingesting ~20 g of dietary protein immediately after exercise seem to provide an optimal dose to stimulate muscle protein synthesis in young adults, whereas older adults may need greater doses [18;19]. In addition, repeating the bolus regularly every 3 h after exercise may further stimulate the protein synthesis post-exercise [20]. However, the effect of regular team sport training combined with protein supplementation on muscle mass remains unknown in older adults.

Thus, the aim of the present study was in untrained old adults to examine the effect of small-sided team sport ball games followed by high or low protein intake on muscle mass, muscle strength, physical function and physiological adaptations important for health, including inflammatory status, blood lipid profile, and heart rate at rest and during exercise. It was hypothesized that regular team sport training with high or low protein intake in the older subjects would lead to a broad range of favorable adaptations including better physical function and muscle strength, lower heart rate at rest and during exercise as well as a drop in plasma low-density lipoprotein (LDL) cholesterol, triglycerides and C-reactive protein (CRP) compared to control. Furthermore, that ingesting protein after small-sided ball game training was necessary to obtain gain in muscle mass in untrained older adults.

## Methods

### Deviations from the study protocol

The present study was a part of an interdisciplinary project, covering multiple physiological, sociological and psychological research aims. Thus, only a part of the study protocol is covered in the present manuscript, and the main study is published elsewhere [21]. For information about included sections and topics in the present randomized trial, please see the CONSORT checklist (S1 File). In short, major deviations from the study protocol (S2 File and S3 File) includes (I) a three-group study design with two intervention groups and one control group instead of a two-group design, (II) two test rounds conducted before and after a 12-week intervention period without the described test rounds after 6 and 12 months, (III) a statistical approach using analysis of covariance (ANCOVA) including the three groups as a categorical independent variable while adjusting for baseline values of the outcome, age and gender instead of the described analysis of variance (ANOVA). For further details, please see *design* and *statistics*. For practical reasons, the present study was registered at ClinicalTrials.gov (trial number: NCT03120143) after participant enrolment. The authors confirm that all ongoing and related trials for this intervention are registered.

### Subjects

**Fig 1:**
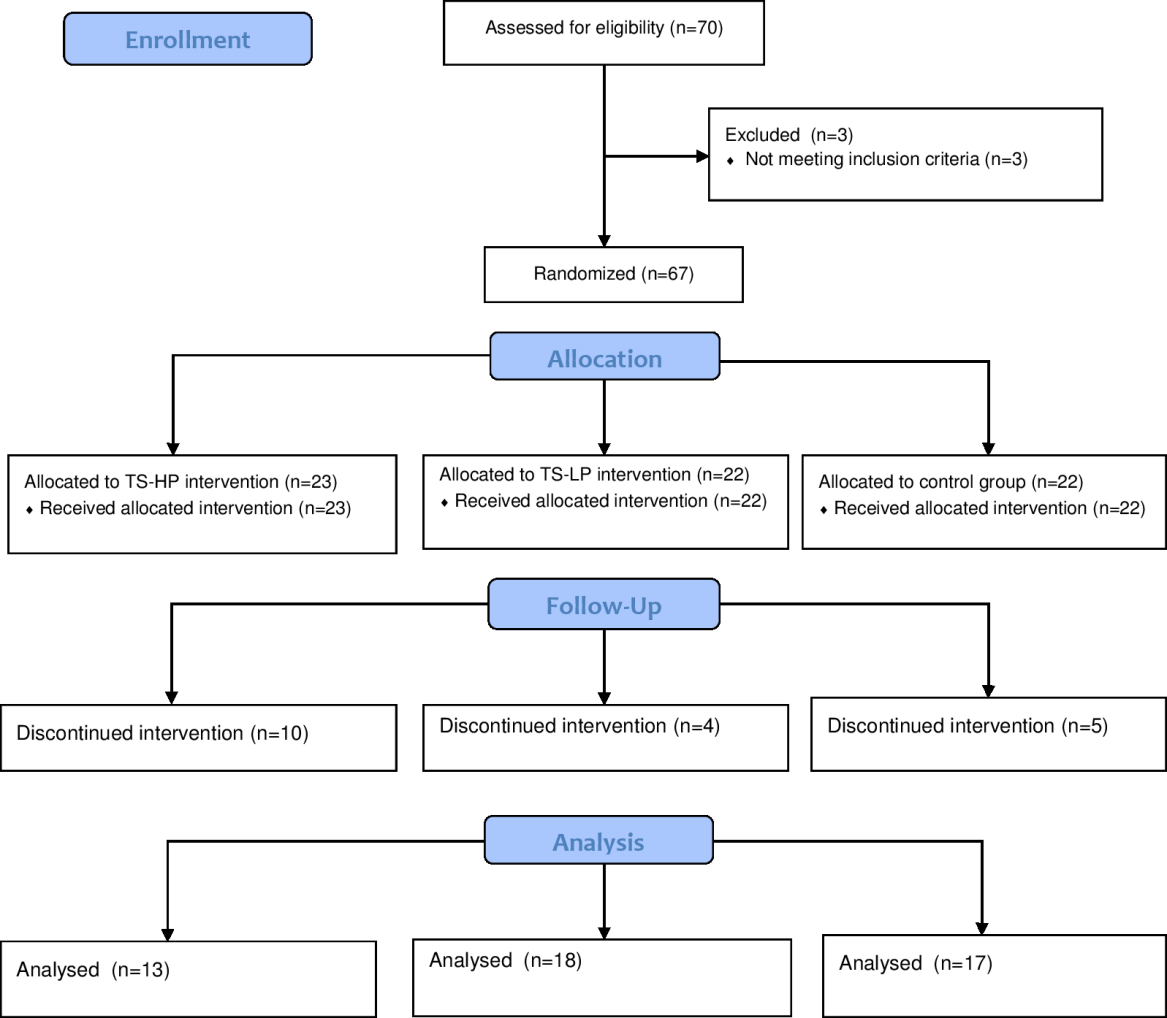
Consort flow diagram of the three study groups. TS-HP, the team sport and high protein group; TS-LP, the team sport and low protein group.

Sixty-seven untrained older men (n = 28) and women (n = 39) aged 72±6 (range: 65–90) years were recruited to the study in the period January to March 2016 through newspaper advertisements and municipal-based centers for older citizens (Table 1 and Fig 1). Exclusion criteria included severe cardiovascular or neurological disease and participation in regular intense exercise training or sports. Participants were recreational active on a daily basis, i.e. walking and cycling in leisure time and for transportation, but were not involved in any structured sport or training activities on a regular basis. In accordance, accelerometer measurements showed that the most prevalent daily physical activity was walking (~1 h) and the daily number of steps were ~7400 which places this group in the highest quintile in older adults aged 70–75 years with regard to steps per day [22], whereas weekly running time was negligible (<1 min). 48 participants completed the study of which 26 regularly took medicine to lower blood pressure (n = 22), cholesterol (n = 3), or for treatment of thyroid disorder (n = 4). These participants maintained their intake of medicine during the intervention period. The intervention period was conducted in the period March to June 2016 and registered at clinicaltrials.gov in April 2017. The authors confirm that all ongoing and related trials for this intervention are registered. The study was approved by the local ethics committee of Copenhagen (H-15002409) and conducted in accordance with the guidelines of the declaration of Helsinki. The subjects were informed of any risks and discomforts associated with the experiments before giving their written informed consent to participate in the study.

**Table 1:** Participant characteristics and daily energy and macronutrient intake in the team sport and high protein (TS-HP), team sport and low protein (TS-LP) and the control group (CON) before the intervention period.

	TS-HP	TS-LP	CON
N	13	18	17
Male, n	6	8	8
Female, n	7	10	9
Age (years)	69±3	74±6	72±7
BMI (kg/m^2)	26±4	28±4	25±5
Energy intake (kJ)	8443±2229	7076±1402	7791±2721
Protein (g)	87±23	66±11	69±21
Protein (g/kg)	1.2±0.3	0.9±0.2*	1.0±0.3
Carbohydrate (g)	204±76	179±59	185±96
Fat (g)	79±21	68±18	67±28

Means ± SD are presented.

*Significant different from CON.

### Design

The participants were matched by their performance in a 2.45-m up-and-go test (see below) and randomized to either a team sport group ingesting a drink high in protein (18 g; TS-HP, n = 13) or an isocaloric drink with low protein content (3 g; TS-LP, n = 18) immediately and 3 h after each training session, or a control group continuing their habitual lifestyle (CON, n = 22). Before and after a 12-week intervention period, the participants carried out two experimental days to assess physical function, muscle strength, body composition, blood lipids, systemic inflammation, and heart rate at rest and during exercise. In addition, before and during the intervention period, daily physical activity level during 8 days were recorded by accelerometer measurements and weighed dietary intake was registered for 3 days (see *Measuring and test procedures*). Apart from the training sessions, participants were instructed to continue their habitual diet routines and level of physical activity.

### Training

Training consisted of customized and supervised small-sided floorball and ‘cone ball’ performed indoor on a wooden surface sized either 10x20 or 10x10 m. Floorball is a team sport like hockey, but played with plastic sticks (http://www.floorball.org). Cone ball was performed with a soft ball and six cones in each end of the playing area. Scoring was achieved by successfully hitting one of the cones of the other team. No movement with ball was allowed, but the other participants could move freely. The floorball and cone ball games were played 3 *vs*. 3 or 4 *vs*. 4, and no physical contact was allowed. During the first 4 weeks, participants performed 4x4 min separated by 4 min of rest, and this was progressively increased to 5x4 and 6x4 min in weeks 5–8 and 9–12, respectively. Number of floorball and cone ball training sessions was evenly distributed during the training intervention, i.e. 50% of training sessions consisted of floorball and 50% of cone ball. A 10-min warm-up session, including mobility, stretching and technical exercises with the ball proceeded every training session.

### Diet intervention

In TS-HP and TS-LP, an isocaloric drink high and low in protein was ingested immediately after and 3 h following each training session, respectively. If the post 3 h drink overlapped with a main meal, the participants were instructed to ingest the drink 1 hour before or after the main meal. The drink high in protein consisted of 250 mL low fat milk (0.5% fat per 100 ml) mixed with protein powder (Meritene Energis, Nestlé Health Science, Sweden) with a total energy, protein, carbohydrate and fat intake per drink of 853 KJ, 18.0 g, 28.6 g and 1.6 g, respectively. The drink low in protein consisted of 500 mL apple or orange juice (Rynkeby Foods A/S, Ringe, Denmark) with a total energy, protein, carbohydrate and fat intake per drink of 900 KJ, 3.0 g, 47.5 g and 0.5 g, respectively.

### Training and diet compliance

All participants were offered three weekly supervised training sessions during the intervention period and TS-HP and TS-LP trained together. The weekly number of training sessions and compliance were the same in TS-HP and TS-LP (2.2±0.3 *vs*. 2.0±0.4 sessions and 72±9 *vs*. 67±15%, respectively). Subjects with a training compliance <50%, i.e. a weekly training compliance lower than 1.5 times, were excluded from the data (n = 1 from TS-HP). Protein and carbohydrate compliance immediately after a training session was 100% as intake was controlled and supervised. The post 3 h protein and carbohydrate intake was not supervised, but interviews with the subjects after the intervention period indicated that all had complied with the post 3 h protocol.

### Participant drop out and injuries

Forty-eight participants completed the study (TS-HP, n = 13; TS-LP, n = 18; CON, n = 17) and nineteen participants dropped out (TS-HP, n = 10; TS-LP, n = 4; CON, n = 5). The major reason for drop-out was inability to facilitate transportation to the location of training (n = 9), as the location of the training intervention was moved during the second training week to meet the need for larger training facilities. Other reasons for drop-out were dissatisfaction with the randomization (n = 3), injury during training (n = 2), lack of motivation for training (n = 2), lack of time to perform tests (n = 1), gastrointestinal discomfort after protein intake (n = 1), and low training participation (n = 1). All except two participants completed the training without major injuries. Two participants (~6%) suffered an injury during training (one had a shoulder injury after a fall, and one a knee-injury). When minor injuries occurred (e.g. muscle soreness), the participant was instructed to rest for a period (e.g. 3–7 days without training) before resuming training.

### Measuring and test procedures

All experimental days were preceded by at least 36 h of refraining from strenuous exercise. Caffeine ingestion, e.g. through coffee, tea, soft drinks, was not allowed at least 16 h before experimental tests to avoid any possible effect on blood pressure, heart rate and physical performance. To minimize influence of diet, each subject registered a 24-h diet log the day before the first experimental day, and this diet log was replicated before each forthcoming experimental day. Subjects on medicine were instructed to take their habitual medicine as usual on experimental days.

### First experimental day

On the first experimental day, the subjects reported to the laboratory between 8.00 and 11.00 AM after an overnight fast. Water intake was standardized to 400 mL to avoid body water content to influence body composition during dual-energy X-ray absorptiometry (DXA) scanning. Participants rested at least 15 min in a supine position before blood pressure was measured three consecutive times by an automatic upper arm blood pressure monitor (M7, OMRON, Vernon Hills, IL, USA). If the third measurement of the systolic blood pressure was 5 mmHg different from the second, a fourth measurement was undertaken. Blood pressure was determined as the mean of the last two measurements. After the blood pressure measurements, a blood sample was taken from the cubital vein for determination of fasting blood lipoproteins, triglycerides, glucose, insulin and CRP. Body composition was determined by whole-body DXA scanning (Lunar Prodigy Advance; GE-medical Systems, Madison, WI, USA) and analyzed by software (enCORE v15, GE-medical Systems, Madison, WI, USA). Weight was measured by use of a calibrated digital scale.

### Second experimental day

On the second experimental day, subjects reported to the laboratory between 10.00 and 12.00 AM after a self-chosen breakfast. The subjects were told to replicate their breakfast from the pre-test during the post-test in order minimize the effect of diet on physical performance. Three standardized functional exercises were performed including 1) maximal sit-to-stand repetitions in 30 s, 2) time to sit-to-stand interspersed by a 2 x 2.5 m walk back and forth around a cone, and 3) maximal repetitions of biceps-curls in 30 s with an 8 lbs (3.6 kg) and 5 lbs (2.3 kg) dumbbell for men and women, respectively. Maximal thigh strength was determined as maximal isometric voluntary contraction (MVC) sitting in a special designed chair with one ankle strapped to an isometric strain gauge. The highest force output of three trials was used as the test result. If the highest force output was performed in the final attempt, another trial was carried out. A 5 min standardized walking test wearing heart rate monitors (Polar Team System, Polar Electro Oy, Kempele, Finland) was performed to determine heart rate during submaximal exercise. Participants walked on a 43 m long circuit lane marked by cones with the pace being controlled by repetitive audio beeps when one round had to be reached. Mean heart rate over a 3-min period (from 2 to 4 min) was subsequently calculated and used as the test result. All tests were performed indoor on a wooden surface.

### Daily physical activity

Weekly level of physical activity was determined by accelerometer measurements (Axivity, Newcastle, UK). The accelerometer was placed on the thigh, and carried for 8 consecutive days before removal. Acti 4 software was used to discriminate between physical activity types, including sitting, standing, walking, fast walking (more than 99 steps per min), running, cycling, sit-to-stand movements (i.e. transitions from sitting to upright stand), and number of steps based on threshold values of standard deviation of acceleration and the derived inclination [23]. Daily time as well as changes over time (before vs. during intervention) for the above mentioned activity types were calculated. Non-exercise physical activity (NEPA) was calculated by subtracting exercise training from total daily physical activity during the intervention and subtracting activities at the same time interval the same days before the intervention.

### Diet registration

Diet logs were handed out and dietary intake was recorded electronically over three days including two weekdays and one weekend day. In TS-HP and TS-LP, diet recordings included at least one day with training. Total energy, protein, carbohydrate and fat intake were analyzed by use of a dietary software program (DanKost Pro, Copenhagen, Denmark).

### Heart rate response and activity profile during training

At selected training sessions, participants were wearing a heart rate monitor (Polar Team System, Polar Electro Oy, Kempele, Finland) to measure heart rate response during both floorball and cone ball training. Average and peak heart rate as well as the distribution in heart rate zones were determined (Polar ProTrainer 5, Finland). It was not possible to conduct a maximal exercise test for determination of maximal heart rate, however, the study of Vorup et al. (2016) showed that heart rate during floorball training in average reached 95% of maximal heart rate in untrained older men. Thus, in the present study maximal heart rate was estimated as the peak heart rate during any training session multiplied by a factor (100/95).

Video recordings were undertaken to determine the activity profile and number of intense actions of ten participants (men, n = 5; women, n = 5) during training. The recordings were subsequently analyzed (TimeMotion app for iPad, Apple Inc., Cupertino, CA, USA) and the activity profile (total duration of standing still, standing movements, walking, and running) and number of intense actions (rapid de-acceleration, rapid turns 180 degree, rapid changes in direction 90 degrees, shot or throws, overhead arm-stretch, hand under knees) were determined during both floorball and cone ball training.

### Blood analysis

Whole blood samples were analyzed at the clinical biochemical unit at the Copenhagen main hospital (Rigshospitalet) using an automatic analyzer with enzymatic kits for total plasma cholesterol, LDL cholesterol, high-density lipoprotein (HDL) cholesterol, triglycerides, glucose, and CRP by using turbidimetric immunoassay (COBAS 8000, Roche Diagnotics International Ltd, Rotkruz, Switzerland). Blood samples for collection of plasma were drawn into tubes containing ethylenediamine tetraacetic acid (EDTA) as anticoagulant. Blood was then immediately centrifuged for 60 s 1,500 *g*, and plasma was then stored at -80°C for later analysis. Plasma concentrations of insulin were measured using an enzyme immunoassay (Insulin ELISA kit, Dako, Glostrup, Denmark).

### Data treatment

Two participants’ blood samples were excluded from the data, including total plasma cholesterol, LDL cholesterol, HDL cholesterol, triglycerides, glucose, CRP and insulin resistance determined by homeostatic model assessment (HOMA-IR) since it was not possible to analyze the samples (n = 2 from TS-HP). In CRP data, any participants with more than 5-fold changes after compared to before the training were excluded to minimize the risk of any acute stimuli not related to training, e.g. minor infections (n = 1 from TS-HP and n = 1 from TS-LP). In the MVC test, participants with minor leg injuries or muscle soreness unable to perform maximal effort were excluded (TS-LP, n = 2 and CON, n = 1). In the walking test, it was not possible to analyze the heart rate response of four participants due to technical reasons (n = 1 from TS-HP; n = 2 from TS-LP; n = 1 from CON).

### Statistics

Comparisons of two means between groups were performed using a two-tailed unpaired t-test. For the considered outcome measures, including NEPA, the effects of training with high protein or low protein intake were evaluated using analysis of covariance (ANCOVA) including the groups (TS-HP, TS-LP, and CON) as a categorical independent variable while adjusting for baseline values of the outcome, age and gender. Contrast estimates (CE), 95% confidence intervals, and p-values were reported for TS-HP and TS-LP (see *Results*). CE estimates the mean difference compared to CON. Distribution of the data was checked for normality before applying the t-test or ANCOVA. The post-hoc sample size justification considered in the Discussion section was based on contrast estimates in leg muscle mass (0.6 kg) and delta standard deviation (0.6 kg) in TS-HP and CON. IBM SPSS statistics 22.0 was used for all tests. P<0.05 was chosen as the level of significance and all data are presented as means ±standard deviation (SD).

## Results

Contrast estimates, 95% confidence intervals and significance levels based on the ANCOVA model for all outcome measures are shown in Table 2.

**Table 2:** Contrast estimates (CE), 95% confidence intervals (95% CI) and p-values (P) for comparing outcome measures in the team sport and high protein (TS-HP) and team sport and low protein group (TS-LP) to a control group.

	TS-HP			TS-LP		
	CE	[95% CI]	P	CE	95% CI	P
Total muscle mass (kg)	0.9	[-0.2; 2.0]	0.10	0.4	[-0.6; 1.3]	0.48
Leg muscle mass (kg)	0.6	[0.0; 1.2]	0.047*	-0.0	[-0.6; 0.5]	0.97
Arm muscle mass (kg)	0.2	[-0.2; 0.5]	0.35	-0.1	[-0.4; 0.2]	0.18
Total fat mass (kg)	-0.9	[-2.1; 0.2]	0.11	-1.2	[-2.2; -0.1]	0.03*
Abdominal fat mass (kg)	-0.3	[-0.6; 0.1]	0.13	-0.4	[-0.7; -0.1]	0.02*
MVC (N)	-10	[-51; 30]	0.60	-15	[-52; 23]	0.44
30 Sit-to-stand (repetitions)	1.2	[-0.0; 2.5]	0.05	1.6	[0.5; 2.8]	0.01*
2.45 m up-and-go (s)	-0.7	[-1.3; -0.1]	0.03*	-0.4	[-1.0; 0.1]	0.13
Arm curls (repetitions)	3.5	[1.1; 5.9]	0.01*	1.4	[-0.7; 3.5]	0.19
Heart rate at rest (bpm)	-4.2	[-9.9; 1.5]	0.14	-8.7	[-13.9; -3.5]	0.002*
Heart rate walking (bpm)	-2.3	[-8.4; 3.9]	0.46	-4.5	[-10.2; 1.5]	0.12
Systolic blood pressure (mmHg)	-0.4	[-9.0; 8.1]	0.92	4.3	[-3.4; 12.1]	0.27
Diastolic blood pressure (mmHg)	1.6	[-3.3; 6.5]	0.52	-1.6	[-6.0; 2.8]	0.47
Total cholesterol (mmol/L)	-0.1	[-0.4; 0.2]	0.48	0.0	[-0.2; 0.3]	0.80
HDL cholesterol (mmol/L)	0.0	[-0.1; 0.1]	0.91	-0.1	[-0.2; 0.1]	0.35
LDL cholesterol (mmol/L)	-0.2	[-0.4; 0.1]	0.22	0.1	[-0.1; 0.4]	0.32
Triglycerides (mmol/L)	0.0	[-0.2; 0.2]	0.84	0.0	[-0.1; 0.2]	0.79
Glucose (mmol/L)	-0.0	[-0.3; 0.3]	0.96	0.1	[-0.2; 0.4]	0.53
Insulin (pmol/L)	-1	[-8; 7]	0.84	-4	[-10; 3]	0.27
HOMA-IR	0.0	[-0.3; 0.3]	0.98	-0.1	[-0.4; 0.2]	0.55
C-reactive protein (mmol/L)	-1.1	[-3.0; 0.7]	0.23	-1.8	[-3.4; -0.2]	0.03*

BP; blood pressure.

*Significant effect compared to CON, based on the ANCOVA model with adjustment for baseline outcome, age and gender.

### Baseline measurements

At baseline, CRP was lower (P = 0.02) in TS-HP compared to CON (1.0±0.6 vs. 2.3±3.1 mmol/L). No other differences between groups were observed at baseline.

### Heart rate and activity profile during training

In cone ball, number of overhead arm-stretches and hands under knees was higher (P = 0.00001 and P = 0.0004, respectively) compared to floorball, whereas number of shots and throws was lower (P = 0.04; Table 3). No differences in number of rapid de-accelerations, 180 degrees rapid turns and 90 degrees rapid changes in direction were observed between cone ball and floorball (Table 3). Total duration of standing still, standing movements, walking and running was the same in cone ball and floorball (Table 3). Also, mean and peak heart rate in cone ball was similar to the response in floorball (Table 3).

**Table 3:** Activity profile, number of intense actions and heart rate response during floorball and cone ball training.

	Total duration (min)	
	Floorball	Cone ball
Type of activity		
Standing still	1.2±0.9	1.5±0.9
Standing movements	8.4±1.3	7.9±1.2
Walking	7.1±1.3	6.6±2.0
Running	3.3±1.9	3.9±2.3
Total	20.0±0.1	19.9±0.6
	Number of actions/20 min	
	Floorball	Cone ball
Type of intense action		
Fast de-accelerations	36±14	35±14
Fast 180 degrees turns	54±13	56±24
Fast 90 degrees change of directions	41±22	50±19
Overhead arm stretches	1±1	33±11*
Hands under knees	2±1	35±18*
Shots or throws	66±21	48±12*
Total	201±25	256±9
	Total duration (min)	
Heart rate zone (% of maximal heart rate)	Floorball	Cone ball
60–69	13 ± 5	13 ± 5
70–79	13 ± 5	11 ± 4
80–89	14 ± 5	14 ± 5
90–100	4 ± 4	5 ± 4
Peak heart rate (bpm)	143 ± 18	143 ± 19
Mean heart rate (% of maximal heart rate)	72 ± 5	73 ± 5

Means ± SD are presented.

### Daily physical activity

No effect of neither TS-HP nor TS-LP was observed on NEPA during the intervention compared to CON (Table 4).

**Table 4:** Non-exercise daily physical activity in the team sport and high protein (TS-HP), team sport and low protein (TS-LP) and the control group (CON) before and during the intervention period.

	TS-HP		TS-LP		CON	
Activity	Before	During	Before	During	Before	During
Sitting (min)	622±98	614±114	641±92	576±111	594±122	577±142
Standing (min)	156±34	160±33	151±54	159±70	200±83	216±98
Walking (min)	74±29	89±29	57±28	78±26	65±29	78±29
Walking fast (min)	52±20	61±23	42±25	57±22	44±24	49±23
Running (min)	0.2±0.3	0.1±0.1	0.1±0.1	7±29	0.1±0.1	0.1±0.1
Cycling (min)	4±7	9±10	4±10	11±19	3±4	5±7
Sit-to-stand (n)	56±20	66±15	51±20	70±45	54±22	62±27
Steps (n)	8408±3240	10198±3053	6747±3440	10500±4898	7357±3131	8648±3199

Means ± SD are presented.

### Daily diet intake

In TS-HP, daily energy and protein intake during the intervention period was higher (P = 0.05) than in CON (Table 5), whereas no differences were observed in daily carbohydrate and fat intake. No differences in daily energy and macronutrient intake during the intervention were observed between TS-LP and CON (Table 5).

**Table 5:** Daily energy and macronutrient intake in the team sport and high protein (TS-HP), team sport and low protein (TS-LP) and the control group (CON) during the intervention period.

	TS-HP	TS-LP	CON
Energy intake (kJ)	8893±605*	6536±320	7213±537
Protein (g)	91±9*	66±4	75±6
Protein (g/kg)	1.2±0.4	0.9±0.2	1.0±0.5
Carbohydrate (g)	224±25	166±10	197±19
Fat (g)	78±6	59±4	71±7

Means ± SD are presented.

*Significant different from CON.

### Physical function and maximal voluntary contraction

**Fig 2:**
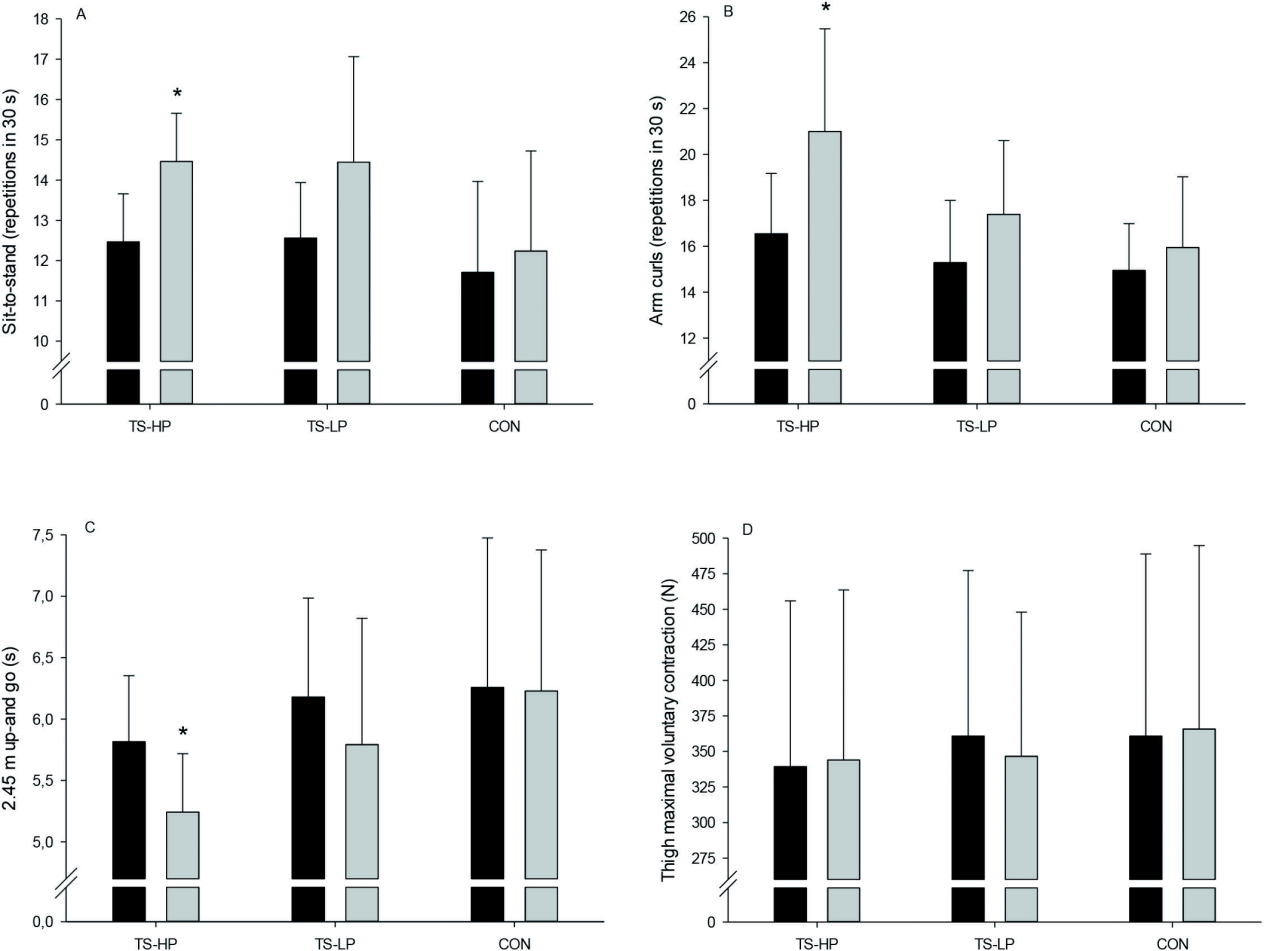
Sit to stand (A), arm curls (B), 2.45 m up-and-go (C), and thigh maximal voluntary contraction (D) before (black bars) and after (grey bars) the 12-week intervention period for the team sport and high protein (TS-HP, n = 13), team sport and low protein (TS-LP, n = 18 except for D, n = 16), and the control group (CON, n = 17 except for D, n = 16). Data are presented as means ± SD. *Significant effect compared to CON, based on the ANCOVA model with adjustment for baseline outcome, age and gender.

In TS-HP, number of sit-to-stand repetitions in 30 s was 1.2±0.6 higher (borderline significant, P = 0.054) after the intervention compared to CON, with an increase (P = 0.01) of 1.6±0.6 in TS-LP (Fig 2A). In TS-HP, number of biceps curls was 3.5±1.2 higher (P = 0.01) after the intervention period compared to CON, with no effect observed in TS-LP (Fig 2B). In TS-HP, time to perform 2.45 m up-and-go was 0.7±0.3 s lower (P = 0.03) after the intervention compared to CON, whereas no effect was observed in TS-LP (Fig 2C). No effect in thigh MVC was observed in neither TS-HP nor TS-LP (Fig 2D).

### Body composition

**Fig 3:**
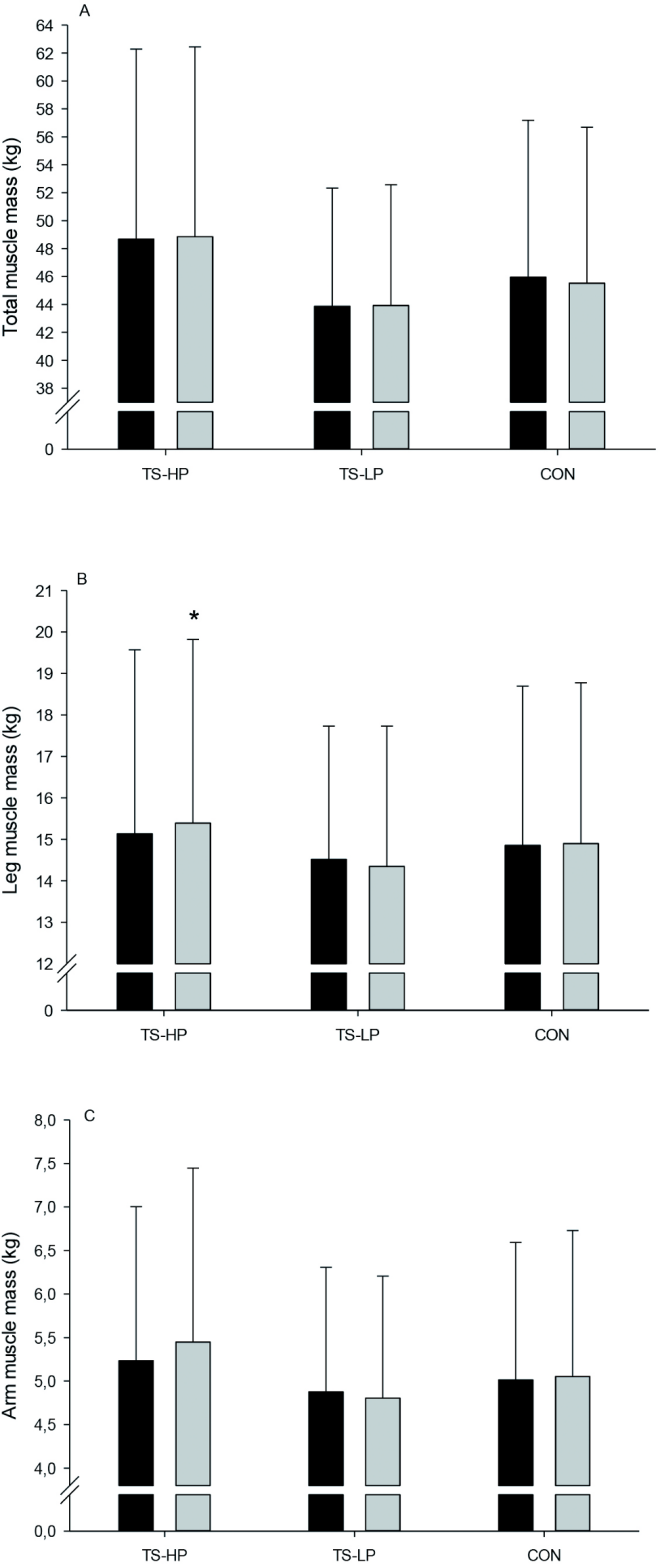
Total muscle mass (A), leg muscle mass (B), and arm muscle mass (C) before (black bars) and after (grey bars) the 12-week intervention period for the team sport and high protein (TS-HP, n = 13), team sport and low protein (TS-LP, n = 18) and the control group (CON, n = 17). Data are presented as means ± SD. *Significant effect compared to CON, based on the ANCOVA model with adjustment for baseline outcome, age and gender.

**Fig 4:**
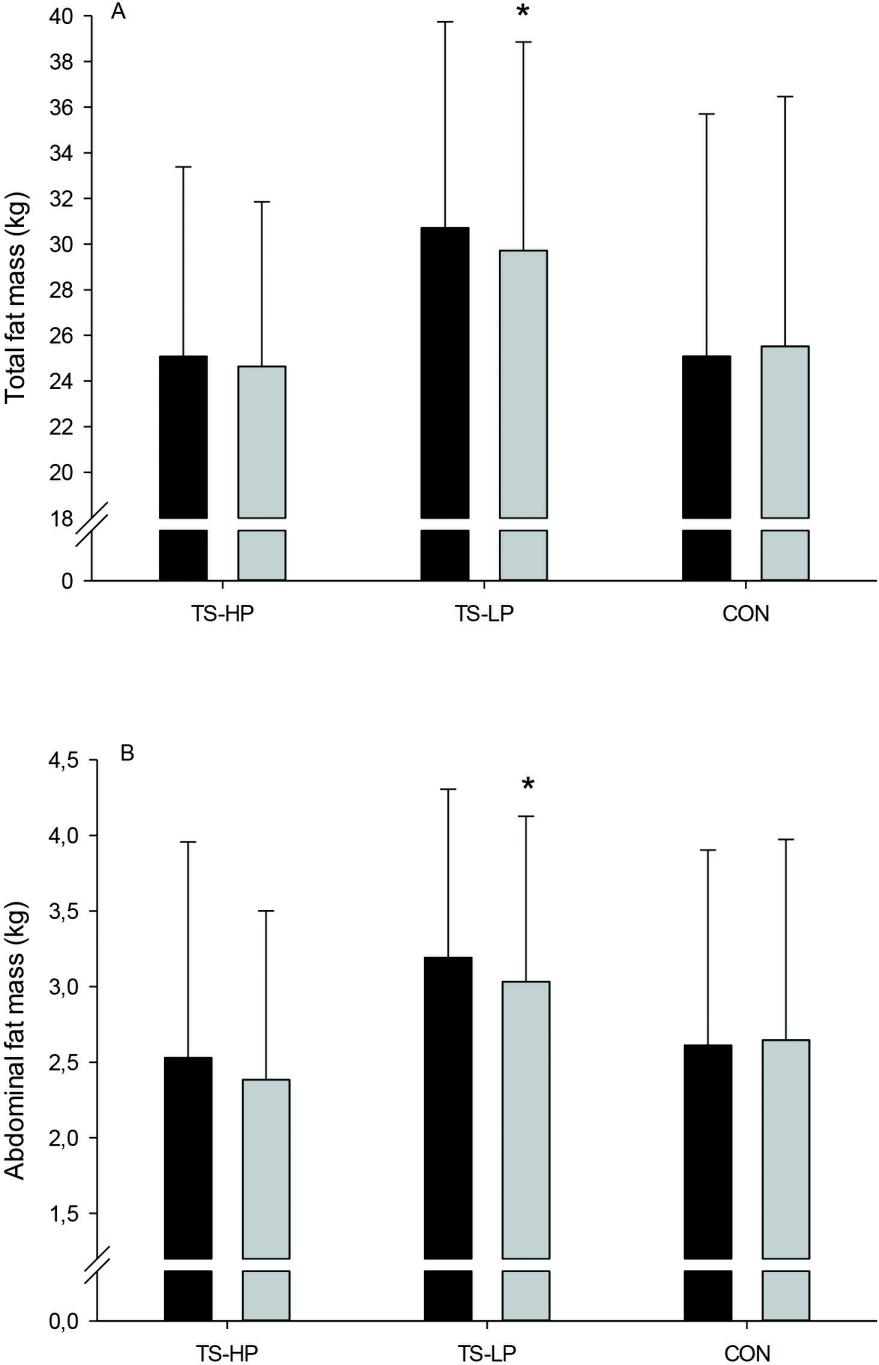
Total fat mass (A) and abdominal fat mass (B) before (black bars) and after (grey bars) the 12-week intervention period for the team sport and high protein (TS-HP, n = 13), team sport and low protein (TS-LP, n = 18) and the control group (CON, n = 17). Data are presented as means ± SD. *Significant effect compared to CON, based on the ANCOVA model with adjustment for baseline outcome, age and gender.

In TS-HP, leg muscle mass was 0.6±0.3 kg higher (P = 0.047) after the intervention period compared to CON, with no effect observed in TS-LP (Fig 3B). In TS-HP, no significant effect was observed on total and abdominal fat mass after the intervention period compared to CON (Fig 4A and 4B), whereas total and abdominal fast mass was 1.2±0.5 and 0.4±0.2 kg, respectively, lower (P = 0.03 and 0.02) in TS-LP (Fig 4A and 4B). No effect on total and arm muscle mass were observed in TS-HP and TS-LP (Fig 3A and 3C).

### Blood variables at rest

In TS-HP, no effect on CRP was observed with the intervention, whereas CRP was 1.8±0.8 mmol/L lower (P = 0.03) in TS-LP (Table 6). Neither TS-HP nor TS-LP had an effect on total plasma cholesterol, LDL, HDL or triglycerides (Table 6). Likewise, no effect on plasma glucose, insulin and HOMA-IR were observed with the intervention (Table 6).

**Table 6:** Blood variables and blood pressure at rest in the team sport and high protein (TS-HP), team sport and low protein (TS-LP) and the control group (CON) before (Pre) and after (Post) the intervention period.

	TS-HP		TS-LP		CON	
	Pre	Post	Pre	Post	Pre	Post
Total cholesterol (mmol/L)	5.4±0.9	4.8±0.9	5.5±1.1	5.1±1.0	5.2±1.1	4.8±0.7
LDL cholesterol (mmol/L)	3.3±1.0	2.8±0.7	3.5±0.9	3.3±0.9	3.2±1.0	3.0±0.7
HDL cholesterol (mmol/L)	1.9±0.8	1.8±0.6	1.6±0.4	1.5±0.3	1.7±0.5	1.6±0.4
Triglycerides (mmol/L)	1.0±0.6	0.9±0.5	1.1±0.5	0.9±0.2	1.1±0.7	0.9±0.4
Glucose (mmol/L)	5.0±0.3	5.1±0.3	5.2±0.4	5.2±0.5	5.5±0.8	5.3±0.7
Insulin (pmol/L)	37±23	33±19	34±28	28±18	35±24	32±22
HOMA-IR	1.5±0.9	1.4±0.8	1.4±1.3	1.2±0.8	1.6±1.2	1.4±1.1
CRP (mg/L)	1.0±0.6	0.8±0.6	3.6±3.1	2.5±2.2*	2.3±2.0	3.1±4.4
Systolic BP (mmHg)	137±24	130±15	139±15	136±16	138±22	131±18
Diastolic BP (mmHg)	81±11	80±9	79±7	76±8	76±10	76±8

Means ± SD are presented. BP, blood pressure.

*Significant effect compared to CON, based on the ANCOVA model with adjustment for baseline outcome, age and gender.

### Heart rate and blood pressure

**Fig 5:**
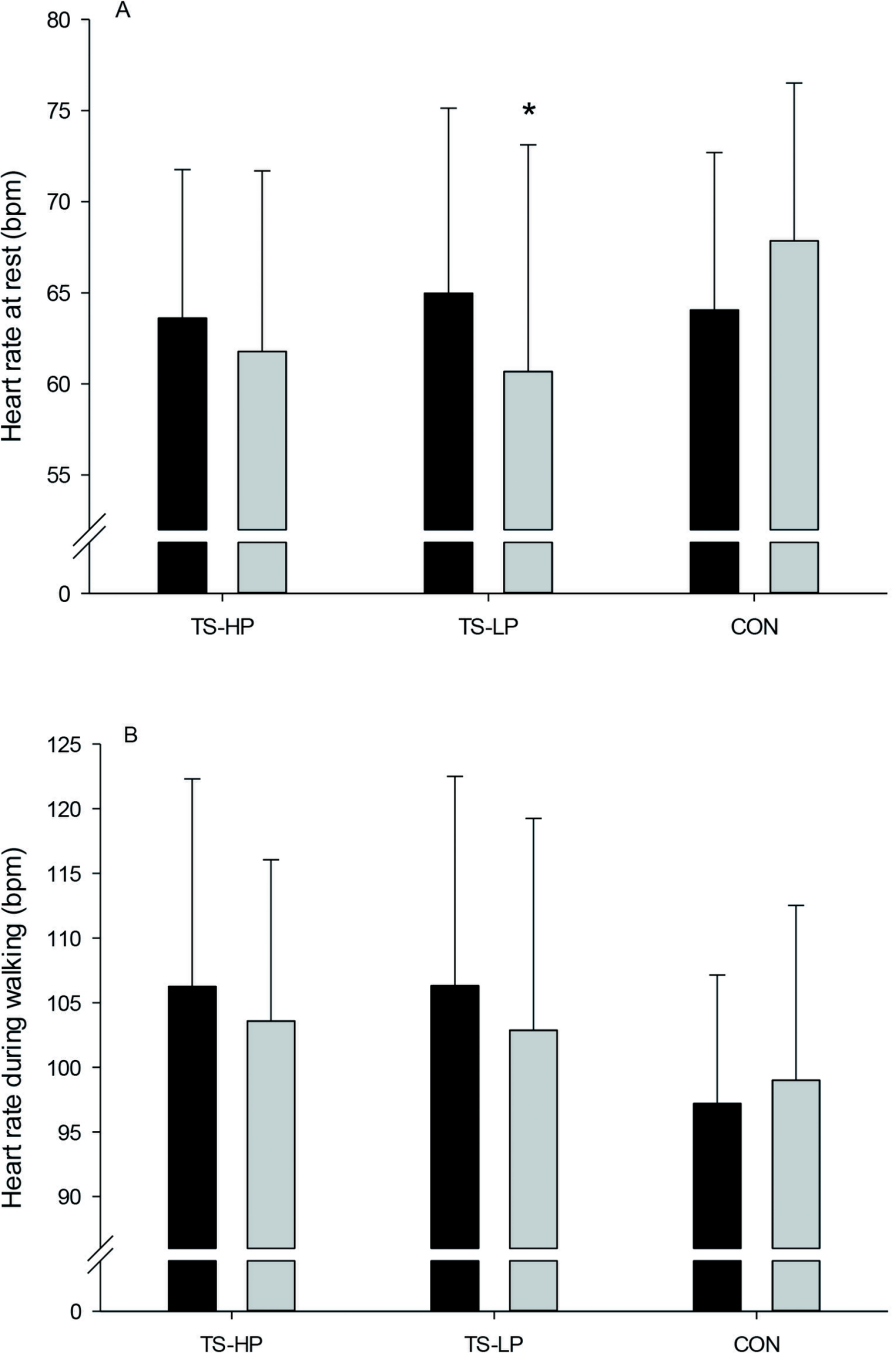
Heart rate at rest (A) and heart rate during walking (B) before (black bars) and after (grey bars) the 12-week intervention period for the team sport and high protein (TS-HP, n = 13 except for B, n = 12), team sport and low protein (TS-LP, n = 18 except for B, n = 16) and the control group (CON, n = 17 except for B, n = 16). Data are presented as means ± SD. *Significant effect compared to CON, based on the ANCOVA model with adjustment for baseline outcome, age and gender.

In TS-HP, no effect on heart rate at rest was observed, whereas heart rate at rest was 9±3 bpm lower (P = 0.002) in TS-LP compared to CON (Fig 5A). No effect on heart rate during walking as well as systolic and diastolic blood pressure at rest were observed with the intervention period in TS-HP and TS-LP (Fig 5B and Table 6).

## Discussion

The main findings of the present study were that small-sided team sport training performed twice a week for ~20 min with intake of 18 g of protein immediately and three h after each session for 12 weeks resulted in higher leg muscle mass and improved 2.45 m up-and-go and arm curl performance in untrained older adults aged 65–90 years. Furthermore, lower abdominal and total fat mass, lower systemic inflammation, and lower heart rate at rest was observed when a drink low in proteins was ingested after the team sport training sessions.

Leg muscle mass increased only in the team sport group that had protein supplementation, which indicates that protein intake after training is important for increases in muscle mass in untrained older adults. In accordance, other studies examining team sport training without protein supplementation in older adults did not find any changes in muscle mass [7;13]. It has been suggested that older adults people have a higher requirement of proteins during a period with exercise training [24]. In the present study, the daily protein intake of the participants was around 1.2 g/kg (~17% of total daily energy intake), which is somewhat lower than the 1.7 g/kg reported in the study of Andersen et al. (2016), but in accordance with the 2012 Nordic Nutrition Recommendations of 1.2 g/kg and higher than the current recommendation of the American Dietetic Association of 0.8 g/kg for older adults. Despite fulfilling daily protein intake recommendations, no change in muscle mass was observed in the group ingesting a low amount of proteins after training. Thus, increased amount of protein after exercise appears to be important in older adult in order to increase muscle mass. In accordance, it has been shown that ingesting protein early after exercise is important for muscle hypertrophy in older adults [25]. Apparently, protein supplementation immediately and three h after exercise only on the training days (~2 weekly sessions) was sufficient to increase leg muscle mass in untrained older adults. In accordance, in older adults it has been found that the protein requirements probably are not increased above normal dietary intake on non-training days as the myofibrillar protein synthesis was found to be similar in an exercised leg 23 h post-exercise whether high-protein (28% of total energy intake) or isocaloric low-protein meals (7% of total energy intake) were ingested [26]. Therefore, when fulfilling the dietary recommendations, timing of protein intake seem to be more important than total protein intake. On the other hand, it cannot be ruled out that the gain in leg muscle mass in TS-HP after the intervention period was a result of a somewhat higher daily protein intake (~1.2 g/kg/day) compared to TS-LP (~0.9 g/kg/day). Nevertheless, although valuable data, the diet recordings should be interpreted with caution since they were based on a random sample from three days only. Another consideration is the statistical power, since post-hoc sample size calculations showed that at least 17 participants were needed to reach sufficient power. In TS-HP, 23 participants were included at the beginning of the intervention period, but were reduced to 13 participants due to drop-out. Thus, future studies should include more participants in order reduce the risk of type I and II errors. Nevertheless, the small-sided ball games were characterized of multiple intense actions, involving considerable eccentric muscle contractions, e.g. during sprints, de-accelerations and rapid turns. Indeed, greater muscle hypertrophy have been associated with eccentric muscle contractions [27], which in combination with the protein supplementation may have provided the stimulus for the higher muscle mass in the legs.

The team sport training combined with high protein intake improved the time to perform 2.45 m up-and-go and number of arm curls within 30 s, whereas the number of sit-to-stand was borderline significant higher (P = 0.054). Better physical function after a period with team sport training was also observed in studies examining small-sided floorball and soccer games in older men aged ~69 years [13;14]. However, compared to the latter studies and adjusted for gender, participants in the present study were slightly older and also had diminished physical function (12 vs. 15–18 repetitions in sit-to-stand, and 6.1 vs. 4.7 s to perform 2.45 up-and-go), showing that the present setup of team sport games also are feasible as well as beneficial in older adults having a lower physical capacity. It is commonly believed that maximal muscle strength and muscle cross-sectional area is strongly related, in particular among older adults, but this association is complex with equivocal findings [28]. In line with this, MVC was not affected by the team sport interventions despite higher leg muscle mass in TS-HP after the training intervention. On the other hand, better physical performance was observed after team sport training, suggesting that higher maximal muscle strength is not necessary for improvements in physical function in older adults. Interestingly, the improvements in physical function were observed despite a relatively small training volume ~20 (range: 16–24) min twice a week. This is advantageous since untrained older adults with a low physical capacity often have problems enduring high training volumes. Both floorball and cone ball were characterized by more than 200 intense actions, including rapid turns and shots with ball, actions that requires coordination, balance and rapid force development. Thus, the intense activity profile of floorball and cone ball training may have provided the stimulus for improvement in physical function.

The period with team sport training combined with a low protein drink led to lowered total and abdominal fat mass. This is in line with observations by Vorup et al. (2016), who found a similar drop in total and visceral fat content after 12 weeks of floorball training in somewhat younger older men (~69 years). Training interventions with small-sided soccer games have also demonstrated decreases in fat mass in young adults [15;29]. The fat loss after team sport training is probably a consequence of the higher total energy expenditure with training, since energy intake was unchanged during compared to before the intervention period. The loss in fat mass after training is probably not related to the post-exercise diet manipulation since the low protein drink was isocaloric with the high protein drink. Indeed, the loss in fat mass in TS-HP and TS-LP was almost similar (0.9 vs. 1.2 kg) compared to CON, but the drop in fat mass in TS-HP did not reach statistical significance (P = 0.11), perhaps because of the limited number of participants in this group (n = 13). Excess body fat, in particular abdominal fat, is associated with a number of comorbidities including insulin resistance and dyslipidemia [30]. Thus, the loss of fat after training may have decreased the risk of developing obesity-related diseases, such as type 2 diabetes and cardiovascular diseases.

CRP was lowered in TS-LP after the intervention period, whereas the drop in CRP in TS-HP did not reach statistical significance. The team sport training may have improved the systemic inflammatory status of the participants as observational studies have showed that a high degree of physical activity and fitness levels are associated with lower plasma concentrations of CRP [31]. On the other hand, the effect of regular exercise on CRP are more ambiguous in randomized controlled trials with most studies showing no effect of regular exercise, including floorball training in untrained older men [13;32;33]. The effect of type, intensity and training volume on CRP is not fully understood and may explain the different outcomes. Furthermore, CRP concentration was more than 3-fold lower in TS-HP compared to TS-LP before the intervention period (1.0 vs. 3.6 mmol/L), indicating that participants in TS-HP were not characterized by systemic low-grade inflammation. Also, a drop in body fat mass, in particular abdominal fat, is associated with decreases in CRP levels possible related to the pro-inflammatory effect of excess body fat [34]. In the present study, the lower CRP in TS-LP was indeed associated with a drop in total and abdominal fat mass, indicating that a part of the lower CRP may be a result of reduced body fat. The lower CRP after training is considered important, since high CRP levels are strongly associated to the incidence of cardiovascular disease [32]. Thus, a 2-fold higher risk of major cardiovascular events exists for persons with CRP levels above 3.0 mg/L compared to levels under 1.0 mg/L [35].

The team sport training with low protein intake led to lower heart rate at rest, probably reflecting an improved cardiovascular capacity. Similarly, it has been observed that heart rate at rest and during submaximal exercise was lowered in a number of studies examining the effect of small-sided soccer or floorball games in young and old adults [12;13]. In the present study, the reduction in heart rate at rest (~4 bpm) was somewhat lower compared to studies examining floorball (~8 bpm) and soccer (~8 bpm) training in untrained older adults [13;14]. This may be related to some differences between the present and latter studies; in the present study the training was apparently less intense illustrated by a lower mean heart rate (~73% vs. 80% of maximal heart rate) and less time spend above 90% of maximal heart rate (~8% vs. 20%). In addition, weekly training volume was lower (~40 min vs. 50 min and 90 min), and the participants were in average slightly older (~72 vs. 69 years) and included participants in the age group 75–90 years. Together, the lower heart rate stimulation during training, the lower total training volume, and perhaps older age may explain that the change in heart rate at rest was relatively modest compared to floorball and soccer training. Post-exercise protein intake is not known to affect heart rate at rest, and it is likely that a higher number of participants in TS-HP may have made it possible to detect a significant change in this group also. Nevertheless, the lower heart rate at rest in the present study is considered important, since the risk for cardiovascular disease does increase with 24% in men and 32% in women with every increase in resting heart rate of 15 bpm [36].

The present study showed that weekly NEPA was not reduced during regular intense small-sided team sport training in old untrained adults, despite the fact that the participants had no previous experience with regular intense training. If any, daily steps and sit-to-stand movements as well as time spend with walking and cycling appeared to increase during the training intervention, and daily sitting time appeared to be lower in the training groups. However, these changes were not significant different from the changes in the control group. Thus, it cannot be concluded that small-sided ball game training results in higher daily activity level. Instead, other variables not related to training, e.g. transition from winter to spring, probably influenced the level of daily activities. Yet, NEPA was maintained during the training intervention which is considered important since low levels of daily physical activity and high sitting time are associated with an elevated risk of cardiovascular disease and metabolic syndrome with the association between sitting time and cardiovascular disease being independent of physical activity level [37–39].

In the present study, no effect of the training was observed on blood lipids and HOMA-IR. Although lower levels of blood lipids are not always found after training interventions [15;40], the finding is in contrast to a marked lowering in LDL, triglycerides and HOMA-IR after 12 weeks of floorball training in older men [13]. It is possible that the weekly training volume in the present training intervention with floorball training and cone ball (~40 min) was too small to induce significant changes in blood lipids and HOMA-IR. On the other hand, a change may not have been expected since much higher weekly training volumes (~90 to 150 min) typically are reported in order to induce significant changes in insulin resistance, plasma LDL and triglycerides [41]. Another explanation may be that metabolic changes of training were blunted due to post-exercise carbohydrate intake, which has been shown to reduce fat metabolism and insulin sensitivity probably related to glycogen re-synthesis status [42;43]. Thus, the immediately and 3 h post-exercise intake of carbohydrate that occurred in both TS-HP (~29 g) and TS-LP (~48 g) may have counteracted metabolic training adaptations that perhaps otherwise would have been seen with calorie restriction, i.e. low glycogen levels post-exercise, but this warrants further investigations.

In summary, higher leg muscle mass after team sport training was most likely a result of post-exercise protein ingestion or perhaps a higher daily protein intake. Furthermore, team sport training in older adults led to a range of important adaptations, including improved physical function when post-exercise protein intake was high, and lower heart rate at rest, reduced CRP levels and a drop in fat mass when post-exercise protein intake was low. Thus, team sport training appears to be recommendable as a health promoting activity, and ingesting protein in combination with team sport training may aid to increase muscle mass in untrained older adults.

## Perspectives and practical considerations

In the present study, 12 weeks of small-sided ball games led to a number of beneficial physiological adaptations in older adults aged 65–90 years despite no previous experience with such training. Importantly, participants were highly motivated, evidenced by 23 of 31 continuing the training after the intervention period, and a low injury rate was observed (~6%). Some key practical aspects may have contributed to the successful intervention: 1) an easy and progressive start-up period and a small training volume per session (range: 16–24 min), 2) games consisted of intervals separated by recovery periods on small-sided areas with few participants, 3) no physical contact was allowed, and 4) a proper warm-up. Furthermore, the present study showed that protein ingested immediately and 3 h after small-sided ball games can increase leg muscle mass in untrained older adults. On the other hand, no changes were observed in blood lipids and insulin. Future research should investigate whether these training adaptations is blunted with carbohydrate intake after exercise. Thus, ingesting food sources high in protein but low in carbohydrate after small-sided ball games may also lead to gains in muscle mass in untrained older adults without compromising adaptations in blood lipids and insulin.

## Supporting information

S1 File CONSORT checklist. (PDF)

S2 File Study protocol in Danish. (PDF)

S3 File Study protocol in English. (PDF)
