# Supplementary material for: Effect of small-sided team sport training and protein intake on muscle mass, physical function and markers of health in older untrained adults: A randomized trial
Source: PLoS One. 2017 Oct 10;12(10):e0186202. doi: 10.1371/journal.pone.0186202 (PMC5634648; doi:10.1371/journal.pone.0186202)
Supplement: S2 File — (PDF) [file pone.0186202.s002.pdf]

København d. 20. januar 2016

Forsøgsprotokol_version 9_20. januar 2016

Anmeldelse til Videnskabsetisk Komité

De Videnskabsetiske Komitéer for Region Hovedstaden

Regionsgården

Kongens Vænge 2

3400 Hillerød

Betydning af holdspilstræning med proteinindtagelse for muskelmassebevarelse, sundhedsfremme, funktionel arbejdsevne samt livskvalitet hos ældre borgere i kommunale aktivitetscentre

Projektbeskrivelse

Formål

Det overordnede formål er at undersøge, hvilken effekt holdspilstræning kombineret med proteinindtagelse har på bevarelsen af muskel- og knoglemasse, ændringer i general sundhedsprofil og den funktionelle arbejdsevne hos ældre borgere over 65 år i kommunale aktivitetscentre. Herunder undersøges det, hvorledes holdspil påvirker den inflammatoriske profil samt hvordan denne relaterer sig til ændringer i muskel- og knoglemasse, funktionel arbejdsevne og sundhedsmål.

Hertil udforskes forsøgsdeltagernes sociale og idrætsmæssige baggrunde, deres formål med at deltage, deres oplevelser med at deltage og deres evaluering efter interventionen. Der vil være et specifikt fokus på om holdspil kan være med til at forbedre deltagernes livskvalitet, selvopfattet fysisk og psykisk helbred, og om det kan styrke gruppesammenholdet samt forebygge frafald i idrætsdeltagelse.

Hypoteser

Det er projektets hovedhypoteser, at holdspilstræning med proteinindtagelse hos ældre borgere over 65 år vil lede til, (I) favorable ændringer i kropssammensætningen i form af tab af kropsfedt, herunder visceral fedt, samt vedligeholdt eller øget muskelmasse, (II) gunstigere lipidprofil i form af sænkning af blodtriglycerider samt højere HDL/LDL kolesterol forhold, (III) øget knogledensitet, (IV) forbedret funktionel arbejdsevne (muskelstyrke, reaktionshastighed og mobilitet, gangudholdenhed, balance samt sidde-stå funktion), (V) reduceret niveau af inflammationsmarkører blodet. Tillige er hypotesen, at holdspilstræning vil føre til øget (VI) fastholdelse i fysisk aktivitet, (VII) selvopfattet helbred og psykisk helbred, (VIII) fordybelse og nydelse ved aktiviteten, (IX) sammenhold, samt (X) højere motivation til at deltage i aktiviteten.

Baggrund

Tab af muskelmasse er aldersrelateret og accelereres fra omkring 65 års alderen (Lexell et al. 1988; Frontera et al. 2000b), hvilket er ledsaget af et betydeligt fald i muskelstyrke (Rosenberg 1997). I takt med stigende muskelatrofi, reduceres både den maksimale muskelkraft samt evnen til at udvikle hurtig kraft (Frontera et al. 2000a; Jespersen et al. 2003), og dette er associeret med et betydeligt tab af funktionsevne, såsom tab af balance, at kunne gå op af trapper, rejse sig op fra en stol og afværgelse af fald (Bean et al. 2002; Cuoco et al. 2004; Foldvari et al. 2000; Holviala et al. 2006; Pijnappels et al. 2005; Skelton et al. 2002; Whipple et al. 1987). Ydermere fører tab af muskelmasse til en betydelig nedgang i evnen til at udføre daglige gøremål samt en øget risiko for fald og frakturer, hvilket svækker uafhængigheden hos ældre (Faulkner et al. 2007). Tab af knoglemasse er også aldersrelateret og accelereres især efter overgangsalderen hos både mænd og kvinder (Kanis & Adami 1994; Beck & Snow 2003), hvilket er vist at være relateret til antallet af knoglefrakturer samt fald hos ældre personer. Tilsammen leder disse faktorer til en øget risiko for sygdom i kredsløb, muskulatur og knogler, der bl.a. manifesterer sig i en øget incidens af type II diabetes, forhøjet blodtryk, knogletab, og forringet postural balance.

Tab af muskelmasse og funktionsevne hos ældre er også en samfundsøkonomisk udfordring. Således skønnes det, at motions- og rehabiliteringstiltag af 6-8 ugers varighed kan sparre en dansk kommune for hjemmehjælpstimer svarende til 13 millioner kr. årligt (DSI 2011) og alene i USA beløber de årlige sundhedsomkostninger relateret til tab af muskelmasse og funktion sig til ca. 20 milliarder dollars (Janssen et al. 2004). I 2004 så man omtrent 41.000 henvendelser på de danske skadestuer, der var relateret til

faldulykker hos ældre (65+ år), heraf førte ca. 12.000 til en indlæggelse (NIPH 2005). Forebyggelse af nedsat bevægelsesfunktion vil således ikke alene være af stor personlig værdi for den enkelte ældre, men også have høj samfundsøkonomisk værdi.

Hos ældre personer der træner regelmæssigt, ser man en forebyggende effekt mod aldersbetinget tab af muskelmasse (Bickel et al. 2011), men de præcise mekanismer hertil er ukendte. Man kan også se, at fysisk aktivitet delvist forhindrer et betydeligt tab af styrke, hvilket kan lede til et vedligeholdt eller forbedret funktionsniveau hos ældre, og denne effekt eksisterer på trods tilstedeværelsen af andre helbredsmæssige problemer (Greig et al. 1993; Rantanen et al. 1997). Effekten har vist sig at være størst hos ældre, der regelmæssigt udførte fysisk aktivitet ved høj intensitet sammenlignet med dagligdagsaktiviteter ved lavere intensiteter (Brach et al. 2004). Det er uklart, hvilke underliggende årsager der bidrager til at modvirke aldersbetinget tab af muskelmasse hos ældre i forbindelse med fysisk aktivitet. De senere år er der dog kommet mere fokus på den mulige betydning af inflammatoriske mediatorer for udviklingen af det aldersrelaterede muskeltab (Beyer et al. 2012a; Roubenoff 2003; Beyer et al. 2012a; Meng & Yu 2010a). Det skyldes bl.a. at plasmaniveauet af TNF-alfa (Tumor Necrosis Factor α), andre proinflammatoriske cytokiner (IL-6, IL-1) og markører for inflammation (CRP, C-Reactive Protein) er vist at være øget ved stigende alder (Beyer et al. 2012b; Meng & Yu 2010b) og ikke mindst, at der er fundet at være korrelation mellem højt serum niveau af pro-inflammatoriske cytokiner og lav muskelstyrke (Visser et al. 2002). Det er dog endnu ikke afklaret om det forhøjede TNF-alfa niveau, der ses hos ældre personer skyldes aldring, kronisk sygdom eller inaktivitet. Derimod synes det sikkert at TNF-alfa har en katabol effekt på skeletmuskelvæv (Schaap et al. 2006) og kronisk inflammation er i øvrigt også blevet associeret med metabolisk syndrom, type II diabetes og åreforkalkning (Petersen & Pedersen 2005; Han et al. 2002; Freeman et al. 2002; Barzilay et al. 2001; Duncan et al. 2003).

Et suboptimalt proteinindtag blandt ældre er associeret med øget risiko for sarkopeni (Houston et al. 2008). Samtidig peger studier på at opmærksomhed på et øget proteinindtag hos ældre, eller et specifikt tilskud af essentielle aminosyrer, herunder bl.a. den forgrenede aminosyre leucin, kan inducere et anabolsk respons i muskel-proteinsyntesen efter træning, der er sammenlignelig med det, der opnås hos yngre mennesker (Moore 2014). Studier blandt utrænede ældre peger desuden på at det er afgørende at det proteinrige måltid indtages kort tid efter træning, for at opnå størst effekt på muskelmassen (Esmarck et al. 2001).

Indtil i dag har forskningen relateret til sundhed og arbejdsevne overvejende været centreret omkring traditionelle motionstyper, som løb og cykling (Cornelissen & Fagard 2005; Pedersen & Saltin 2006) og

fokus har overvejende været på styrketræning i behandlingen af aldersrelateret tab af muskelmasse (Johnston et al. 2008). Nye undersøgelser peger dog i retning af, at motionsformer med et individuelt præg, eksempelvis styrketræning og løb, i mindre grad virker fastholdende sammenlignet med sociale aktivitetsformer så som holdspil (Krustrup et al. 2010b). Tillige viser der sig et generelt tab af livskvalitet, samt selvopfattet fysisk og psykisk heldbred som alderen øges (Bjørner et al. 1997). Det er derfor nødvendigt at undersøge om en social motionstype som holdspil, der afviger fra de traditionelle individualistprægede motionsformer, kan bidrage til at modvirke alderens effekt på disse faktorer, som må siges at være vigtige livet igennem. I denne kontekst er det vist, at fodboldtræning og holdspil kan lede til en række positive psykiske (Elbe et al. 2010) og sociale effekter (Ottesen et al. 2010), herunder større sammenhold (Carron et al. 2002), større fordybelse i aktiviteten (19) og højere motivation til at deltage i aktiviteten (Pelletier et al. 1995). Hertil viser en undersøgelse af inaktive danskere, at centrale motivationsfaktorer for deltagelse i fysisk aktivitet udgøres af positive sociale oplevelser og tidligere idrætsaktivitet (Ottesen og Skjerk 2006).

Nyere dansk forskning har vist, at motionsfodbold kan forbedre sundheden og arbejdsevnen (Krustrup et al. 2010a; Helge et al. 2010; Krustrup et al. 2010c). Det er interessant, at en gruppe ældre utrænede mænd i alderen 63-74 år udviste samme positive forbedringer i funktionel arbejdsevne som en styrketræningsgruppe efter 16 uger med motionsfodbold på små baner (Andersen et al. 2014). Herudover er det vist, at motionsfodbold på små baner kan forbedre den posturale balance (Jakobsen et al. 2011). På nuværende tidspunkt mangler der dog information om effekterne samt gennemførligheden af (I) andre tilpassede holdspil end motionsfodbold, (II) holdspilstræning på funktionssvage ældre, herunder kvinder, samt (III) holdspilstræning på livskvalitet, herunder socialt og mentalt velbefindende.

Samlet set er der et stort behov for at undersøge holdspilstræning som et middel til at fremme både muskel- og knoglebevarelse, funktionel arbejdsevne samt livskvalitet. Studiet kan ligeledes bidrage med vigtig viden om indflydelsen af inflammationstilstande på sundhedstilstanden og funktionalitet hos ældre borgere. Dette studie adskiller sig tillige fra tidligere holdspilstræningsstudier ved at blive afviklet på en samfundsmæssig platform, der muliggør brobygning mellem kommunale aktivitetscentre (Københavns Kommune) og det etablerede frie foreningsliv (DGI).

Design

Studiet er et randomiseret træningsstudie i kommunale aktivitetscentre med holdspil som intervention i 12 måneder hos 80 utrænede mænd og kvinder i alderen 65+ år. Der fordeles 40 til en holdspilsgruppe samt

40 til en kontrolaktivitet i form af styrketræning, som allerede udbydes i aktivitetscentrene. Forsøgsdeltagerne rekrutteres via Københavns Kommunes aktivitetscentre (uddybes i *rekruttering af forsøgspersoner)*. Holdspilsgruppen træner 2-3 gange om ugen a' 16-24 minutter under interventionsperioden og der trænes indendørs eller udendørs via småspil bestående af forskellige tilpassede holdspil (uddybes i *holdspilstræning).* Træningen foregår på udvalgte kommunale aktivitetscentre i København, hvor de rekrutterede brugere af centrene i forvejen har sin regelmæssige gang, hvilket sikrer, at forsøgsdeltagere ikke belastes med yderligere transportbelastning til træningsdestinationen. Der udføres undersøgelser før interventionsperioden samt efter 3, 6 og 12 måneder. Blodprøvetagninger og DXA scanning vil være forbeholdt undersøgelsesrunderne før og efter 12 uger. Således vil undersøgelsesrunderne efter 6 og 12 måneder udelukkende have karakter non-invasive tests, det vil sige interviews, spørgeskemaudfyldelse samt bestemmelse af funktionel arbejdsevne.

Forsøgspersoner

Der rekrutteres 80 utrænede mænd og kvinder i alderen 65+ år.

Eksklusionskriterier

(I) Forsøgspersonerne må ikke have alvorlige komplikationer, herunder betydende polyneuropati, cancer, kendt iskæmisk hjertesygdom eller bevægeapparatlidelser, der hindrer deltagelse i holdspil. Dog er velbehandlet hypertension, dyslipidæmi, samt overvægt og andre aldersrelaterede symptomer af kronisk karakter ikke kontraindikationer.

(II) Forsøgspersonerne skal have en rimelig grad af gangmobilitet, dvs. forsøgspersonen skal kunne gå uden afgørende anvendelse af hjælpemidler som kørestol eller rollator.

(III) Forsøgspersonerne må ikke have noget misbrug af nogen art, herunder alkohol (maksimal 21 genstande om ugen) og euforiserende stoffer (rygning er dog ikke et eksklusionskriterium).

Metoder

Der gennemføres i alt 4 testrunder (før interventionsperioden (efter 0 måneder) samt efter 3, 6 og 12 måneder). Blodprøvetagninger samt DEXA scanning foretages kun ved testrunde 0 og 3 måneder. Således vil testrunde 6 og 12 måneder kun bestå af spørgeskemabesvarelse, interviews samt måling af funktionel arbejdsevne. Testrunderne er beskrevet i detaljer nedenfor.

Beskrivelse af testrunde 0 og 3 måneder

Forsøgspersonerne deltager i to testdage, som er adskilt af mindst 48 timer. Deltagerne må ikke have lavet anstrengende fysisk arbejde og drikke alkohol dagen før en testdag, og må ikke drikke alkohol, te, kaffe, eller ryge før undersøgelserne på selve testdagen.

Testdag 1:

På Institut for Idræt og ernæring, Københavns Universitet, Universitetsparken 13, 2. sal, foretages sundhedsundersøgelse med udtagelse af hvileblodprøve og bestemmelse af kropssammensætning med DEXA scanning. På testdag 1 møder forsøgspersonen op om morgenen i fastende tilstand og tilbydes afslutningsvist en let frokost. Varigheden af testdag 1 er ca. 3 timer.

I forbindelse med blodprøvetagningen anlægges venekateter (venflon) i albuebøjningen til udtagelse af i alt 50 ml blod for at kunne evaluere på den tidsmæssige udvikling af kolesteroltal, triglycerider, blodsukker, insulin, HbA1c, højsensitiv CRP, TNF-alfa, IL-6, IL-1beta og IL-10. Der udtages kun blod ved testrunde 0 og 3 måneder, hvorfor der i alt udtages 100 ml blod gennem hele projektperioden for en deltager.

Der foretages en helkrops DEXA-scanning, som er scanning hvor der udsendes svage røntgenstråler, hvorved kroppens sammensætning af forskellige vævstyper måles (fedtprocent, muskelmasse og knogledensitet). En DEXA-scanning gør ikke ondt og er ikke forbundet med nogen form for ubehag.

Testdag 2:

I forsøgspersonens lokale kommunale aktivitetscenter afvikles ”Senior fitness testen” og efterfølgende besvares spørgeskemaer og der foretages interviews. Varighed af testdag 2 er ca. 3 timer.

Senior Fitness Testen er et simpelt testbatteri, der er udviklet til at måle den funktionelle fitness (det vil sige de funktionelle karakteristika, der er af betydning for funktionel mobilitet i de ældre år). Testene måler således muskelstyrke (under + overkrop), aerob udholdenhed, smidighed, og adræthed/dynamisk balance hos alle niveauer af raske ældre, der kan klare dagligdagen ved egen hjælp. Testen viser resultatet på en kontinuerlig skala og kan således dokumentere ændringer over tid i forbindelse med fx en træningsintervention. Testbatteriet kan udføres i "marken" og kræver ikke specielle laboratoriefaciliteter. Testbatteriet er anvendt i en række videnskabelige undersøgelser (Toto et al. 2012; Santos et al. 2012; Purath et al. 2009; Lobo et al. 2011) og må betragtes som en slags standard til vurdering af ældres fysiske kapacitet udenfor laboratorieregi. Nedenstående er en kort beskrivelse af de udvalgte dele af seniorfitnesstestene, som forventes at anvendes:

Rejse-Sætte sig test (styrke i underkroppen)

Antallet af gange personen kan rejse sig fra siddende i stol til fuld oprejst stilling og sætte sig igen i løbet af 30 sekunder med armene foldet mod brystet.

Armfleksion (styrke i overkrop)

Antallet af armflektioner, der kan udføres i løbet af 30 sekunder med en håndvægt i hånden (≈3,63 kg for mænd).

6 minutters gangtest (aerob udholdenhed)

Antallet af meter, som kan tilbagelægges i løbet af 6 minutter på en 46 meter lang bane.

2,45 m up and go test (adræthed og dynamisk balance)

Antal sekunder, det tager at rejse sig fra siddende stilling (i stol), gå 2,45 meter, dreje og vende tilbage til siddende stilling.

Test af smidighed i underkroppen

Siddende på stolekanten skal personen forsøge at nå tæerne på sit udstrakte ben. Man måler antal centimeter plus eller minus mellem fingerspidser og tåspidser. Resultatet er bedst af 2 forsøg.

Test af smidighed i skuldre og arme

Den ene hånd føres over skulderen og føres så langt ned ad ryggen som muligt, mens den anden hånd føres så højt op som muligt med håndryggen mod ryggen (nede fra). Man noter afstanden i centimeter mellem 3. finger på de 2 hænder (plus eller minus). Resultatet er bedst af 2 forsøg.

Ganghastighed ved 10 m hurtig gang og håndgrebsstyrke

For blandt andet at kunne sammenligne med tidligere danske studier måles hastigheden ved 10 meter hurtig gang og håndgrebsstyrke (HGS). HGS måles i kg med den dominante hånd ved brug af et håndgrebsdynamometer (Takei Scientific Instruments Co. Ltd., Tokyo, Japan). Stående med håndledet i neutral position og strakt albue udføres 3 maksimale forsøg med 1 minut imellem hvert forsøg (bedst af 3).

Flamingo test

Forsøgspersonen skal stå på ét ben over en periode på 60 sekunder med det formål at måle postural balance. Der måles antallet af gange forsøgsdeltageren mister balancen i løbet af de 60 sekunder.

Undersøgelser i løbet af interventionsperioden

I forbindelse med udvalgte træningssessioner foretages pulsmålinger ved hjælp af Polar pulsbælter.

Validerede danske oversættelser af to standardiserede spørgeskemaer vil blive brugt til at måle flow (Flow Kurz Skala: Rheinberg et al., 2003) og i hvor høj grad deltagerne har nydt de aktiviteter, de har deltaget i, (Physical Activity Enjoyment Scale: Kendzierski & DeCarlo, 1991), tre gange i løbet af interventionsperioden. Deltagerne vil blive informeret på forhånd som del af rekrutteringen, og besvarelserne er anonyme og fortrolige.

Uddybende information om spørgeskemaundersøgelser

Der vil blive lavet to spørgeskemaundersøgelser med hver gruppe i interventionen – en baselineundersøgelse ved testrunde 0, der kortlægger deltagernes socioøkonomiske status, aktivitetsniveau og erfaring med idræt, samt en evaluerende undersøgelse til sidst i forsøget ved testrunde 12 måneder. Herudover vil validerede danske oversættelser af standardiserede spørgeskemaer blive taget i brug til at måle selvopfattet fysisk og psykisk helbred (SF-12: Bjørner et al., 1997), angst- og depressionsniveauer (Hospital Anxiety and Depression Scale: Snaith & Zigmond, 1994), livskvalitet (Older People's Quality of Life Questionnaire: Bowling, 2009), aktivitetsniveau (International Physical Activity Questionnaire: Craig et al. 2003), og motivation (Sport Motivation Scale: Pelletier et al., 1995), ved henholdsvis testrunde 0, 3, 6 og 12 måneder.

Uddybende information om interviews

Der vil blive foretaget et antal individuelle interviews og fokusgruppeinterviews med deltagere og medarbejdere fra aktivitetscentrene i projektet. Disse er frivillige at medvirke i, og de medvirkende vil være anonyme. Optagelser af interviews vil blive behandlet fortroligt, og eventuelle udsagn fra dem vil blive pseudonymiseret. Formålet med disse interviews er at afdække deltagernes oplevelser af aktiviteterne, deres sammenhold og motivation, og aktiviteternes effekt på deltagernes livssituation.

Beskrivelse af testrunde 6 og 12 måneder

Testrunde 6 og 12 måneder afvikles som beskrevet ovenfor i *beskrivelse af testrunde 0 og 3 måneder* med undtagelse af testdag 1. Det vil sige, at kun *testdag 2* afvikles, således foretages der ikke blodprøvetagninger samt DEXA scanning ved testrunderne 6 og 12 måneder.

Holdspilstræning

Holdspilstræningen bliver gennemført som småspil udendørs eller indendørs i gymnastikhaller eller tilsvarende arealer i eller omkring aktivitetscentrene. Holdspilstræningen udbydes 3 gange ugentligt, men det er ikke et krav, at man deltager alle 3 gange, dog gerne 2 gange. Der kan varieres mellem tre forskellige holdspil, "Hulahopbold", floorball eller "Bedstevolley".

1) Floorball, hvor der spilles 3 mod 3 på små baner (fx 10x15 meter) i intervaller af 4 minutter adskilt af 4 minutters pauseperiode.
2) "Hulahop-bold", hvor formålet er at kaste bolden gennem en hulahopring ved at aflevere til sine holdkammerater. Der spilles 3 mod 3 på små baner (fx 10x15 meter) i intervaller af 4 minutter adskilt af 4 minutters pauseperiode.
3) "Bedstevolley", en slags alderstilpasset volleyball, hvor der kastes en blød bold over et net (eller snor), som forsøges grebet af et modstanderhold. Der spilles 4 mod 4 på små baner (fx 10x15 meter) og der foretages udskiftninger efter et rotationsprincip.

Ved alle holdspil gælder det, at træningsmængden doseres forsigtigt (startende med 16 minutters effektiv spilletid de første 4 uger, 20 minutter de følgende 4 uger samt 24 min de resterende 4 uger). Kropskontakt under småspillene er ikke tilladt, og der følges op på eventuelle gener fra sidste træningssession før træningen påbegyndes. Holdspilstræningen igangsættes og superviseres af aktivitetscentrets træningsfaglige personale.

Proteinindtagelse

Umiddelbart efter samt 3 timer efter holdspilstræningen vil halvdelen af deltagerne indtage en 0,2 L mælkebaseret proteindrik indeholdende 20 g protein. Det vil sige, at deltagerne vil indtage 40 g protein på en træningsdag, som ved en træningsfrekvens på 2-3 gange om ugen vil føre til en ekstra proteinindtagelse på 80-120 g om ugen. Den anden halvdel af deltagerne fra holdspilstræningen vil indtage en 0,5 L isokalorisk placebodrik bestående af juice umiddelbart efter træning samt 3 timer efter.

Styrketræning

I styrketræningsgruppen deltager man som forsøgsperson i den styrketræning, som udbydes af det lokale aktivitetscenter og træningen udbydes 3 gange om ugen. Det er ikke et krav, at man deltager alle 3 gange om ugen, men gerne mindst 2 gange. Som udgangspunkt udføres 3 øvelser i maskiner ved hvert træningspas. 1) Benpres, 2) skulderpres, og 3) armtræk. Ved hver øvelse gennemføres 3x8 RM (Repetetion Maximum; angiver at belastningen indstilles således, at der kun netop kan løftes 8 gentagelser) og belastningen justeres løbende gennem forløbet. Mellem hvert sæt holdes 2-3 minutters pause. Styrketræningen superviseres af aktivitetscentrets træningsfaglige personale.

Oprettelse af forskningsbiobank, håndtering af biologisk materiale og personoplysninger mv.

Formålet med forskningsbiobanken er at samle og opbevare biologisk materiale til analyser med henblik på opklaring af projektets hypoteser. De udtagne blodprøver (i alt 100 ml. over hele projektperioden) benyttes til en lang række analyser og der vil derfor næppe være noget overskydende biologisk materiale tilbage efter de planlagte analyser. Eventuelt overskydende biologisk materiale vil blive destrueret senest 6 måneder efter projektperiodens ophør. Oplysninger vedrørende forsøgspersonerne beskyttes efter lov om behandling af personoplysninger og sundhedsloven. Hver enkelt forsøgsperson vil efter projektafviklingen have mulighed for at få adgang til egne data samt gennemsnitsdata for hele gruppen. I den skriftlige deltagerinformation spørges om, hvorvidt forsøgsdeltagerne ønsker adgang til egne forsøgsdata senest 2 måneder efter projektafslutning. Alle data vil blive anonymiseret.

Statistiske overvejelser og styrkeberegning

Ændringer i effektparametrene før interventionen bestemmes ved hjælp af en to-halet uparret t-test. Ændringer mellem og indenfor grupperne bestemmes ved en to-vejs ANOVA og lineære regressionsanalyser. Når der detekteres en overordnet signifikant interaktion (tid versus gruppe) eller tidseffekt, anvendes en student-Newman-Keuls post hoc analyse til at isolere hvilken gruppe forandringen fandt sted i.

Antallet af forsøgspersoner er valgt ud fra forventningen om en gennemførelsesprocent på minimum 75%, svarende til minimum 30 deltagere per forsøgsgruppe, og standardafvigelser for forandringerne i effektmålene er fastsat svarende til de observerede ændringer i forskergruppens tilsvarende studier (Krustrup et al. 2009; Krustrup et al. 2010d).

Forventet detektionsgrænse for ændringer i udvalgte main outputs mellem uafhængige grupper (n=30). ANOVA.

Variabel	SD af ændringer	2 grupper
Fedtmasse (kg)	6,1	4,4
Muskelmasse (kg)	2,8	2,1
6-min gangtest (meter)	110	80

Power er sat til 0,8 og P til 0,05

Bivirkninger, risici og ulemper for forsøgspersonerne

I forbindelse med DEXA-scanningerne udsendes svage røntgenstråler, hvorved kroppens sammensætning af forskellige vævstyper måles. Der udsendes 0,0006 mSv ved helkrops-DEXA scanning. Det svarer til en stråledosis, der giver en forøget kræftrisiko på 0,001 %, hvilket må anses for at være en minimal risiko. Den stråledosis forsøgspersonerne får er mindre end ved de fleste røntgenundersøgelser og svarer til 7 dages baggrundsstråling. DEXA-scanningerne gør ikke ondt og er ikke forbundet med nogen form for ubehag.

I forbindelse med blodprøvetagningerne tages blod fra en armvene. Der indlægges et kateter i en vene i albuebøjningen. I forbindelse med fjernelse af kateterne kan der opstå en mindre blødning, der giver misfarvning af huden. For at minimere denne gene vil der blive komprimeret efter udtagelsen (let tryk på stedet). I alt udtages 100 ml. blod (50 ml ved testrunde 0 og 50 ml ved testrunde 3 måneder) i løbet af hele forsøgsperioden på 12 måneder. Til sammenligning udtages 500 ml. ved én enkelt bloddonation. Brug af katetre er benyttet rutinemæssigt på Institut for Idræt og Ernæring, Københavns Universitet, og er brugt uden betydende komplikationer i mere end 30 år.

Risiko ved udførelse af funktionstests i aktivitetscentrene må betragtes som minimal. Alle testene er tidligere udført på ældre, herunder også parkinsonspatienter. Der kan i nogle tilfælde opstå efterfølgende muskelømhed, svarende til almindelig træningsømhed.

Udførelse af holdspilsaktiviteter vurderes, på samme vis som motionsfodbold på små baner, generelt som en sundhedsfremmende aktivitet. Ved at organisere holdspillene som særlig tilpassede småspil (ingen kropskontakt, små baner, få deltagere på hvert hold, indlagte pause mv.) ved man, at risikoen for skader minimeres betragteligt sammenlignet med fx traditionelle fodboldkampe (Krustrup et al. 2010a). Holdspillene superviseres af aktivitetscentrenes træningsfaglige personale. Det er velkendt, at utrænede

individer kan få træningsømhed efter opstart af et træningsforløb. Dette er en naturlig proces, og aftager typisk efter 2-4 uger. Muskelforstrækninger (bl.a. bag- og forlår), overbelastning af sener og vridskader (bl.a. ankler og knæ) kan dog i sjældne tilfælde opstå under holdspilstræning på små baner. Erfaringer fra tidligere holdspilsstudier viser imidlertid, at tilpasset fodbold på små baner (en sammenlignelig aktivitet med holdspilstræning) er en relativ sikker motionsform for utrænede individer, inklusiv ældre, og der rapporteres typisk kun om ganske få skader.

Udførelse af styrketræning vurderes at have minimal risiko, og må betragtes som en sundhedsfremmende og funktionsfremmende aktivitet for ældre individer, hvoraf det også hører til motionsanbefalingerne fra Sundhedsstyrelsen. Styrketræningen finder sted under kontrollede forhold ved brug af dertil indrettede maskiner, hvorved risikoen for skader er minimal. Styrketræningen superviseres af aktivitetscentrenes træningsfaglige personale.

Projektgruppen og initiativtagere

Projektet udgår fra Institut for Idræt og Ernæring samt Center for Holdspil og Sundhed, Københavns Universitet. Initiativtager til projektet er Professor Jens Bangsbo og Københavns Kommune i samarbejde med Danske Gymnastik- og Idrætsforeninger (DGI). Den videnskabelige projektgruppe udgøres af professor Jens Bangsbo, Professor Gertrud Pfister, lektor Mogens Theisen Pedersen, Ph.d. stud. Jacob Vorup, AC-medarbejder Pia Sandfeld Melcher, forskningsassistent Johan Michael Wikman og forskningsassistent Anne Nistrup, alle fra Institut for Idræt og Ernæring, Københavns Universitet. Projektet udføres i samarbejde med Københavns Kommune og DGI.

Økonomisk støtte

Projektet er fuldt finansieret, hvoraf 2/3 af projektet finansieres af Københavns Kommune, mens sidste 1/3 del finansieres af Center for holdspil og Sundhed, Københavns Universitet. Projektmidlerne er udbetalt til en forskningskonto på Institut for Idræt og Ernæring, Københavns Universitet og bruges dels til indkøb af udstyr, til blodanalyser, samt til aflønning af forskningsassistent. Der er tillige afsat midler til overhead. Ingen i projektgruppen har interessekonflikter.

Vederlag til forsøgspersoner

Der udbetales ikke et økonomisk vederlag til forsøgsdeltagerne. Dog dækker projektgruppen nødvendigt udstyr og materialer til afvikling af holdspilstræningen (fx bolde, floorballstave og mål mv.). Ligeledes dækker projektgruppen eventuelle transportudgifter forsøgspersonerne måtte have til transport i forbindelse med testning på Institut for Idræt og Ernæring.

Formidling af forsøgsresultater

Projektets resultater skal formidles bredt og kan allerede fra foråret 2016 løbende indgå i den samfundsmæssige debat via en række af foredrag, konferencer samt andre arrangementer i kommunalt- og foreningsregi. Resultaterne skal formidles både udadtil, evt. via pressekonference eller anden mediebaseret offentliggørelse, samt internt i danske kommuner og i idrætsforeninger, fx gennem DGI. Projektets resultater forventes ydermere at indgå i en række populærartikler, fx i Ældresagen, DGI's magasin UDSPIL og andre relevante tidsskrifter. Det er tillige planlagt at resultaterne skal offentliggøres i forbindelse med en række sundhedskonferencer, hvor fremtidens kommunalt-baserede sundhedsfremmende indsats er til debat. Såvel positive, negative samt inkonklusive resultater vil blive offentliggjort.

Forsikringsforhold

Samtlige deltagere i forsøget er omfattet af patientskadeforsikringen indenfor H:S.

Etiske overvejelser og respekten for forsøgspersonernes fysiske samt mentale integritet

Forsøget godkendes af den videnskabsetiske komité. Holdspilstræning og styrketræning anses for at være træningsforbedrende og generelt sundhedsfremmende for de rekrutterede forsøgspersoner. Blodprøver (højest 50 ml blod ved hhv. testrunde 0 og 3 måneder, svarende til ca. 1 % af den samlede blodmængde) bruges til at afklare projektets problemstillinger og anses ikke som noget etisk problem for en gruppe af ældre personer 65+ år.

Forsøget udføres i overensstemmelse med Helsinki–deklarationen og forsøget anmeldes til Datatilsynet. Oplysninger vedrørende forsøgspersonerne beskyttes efter lov om behandling af personoplysninger og sundhedsloven. Eventuelt overskydende biologisk materiale vil blive destrueret.

Afgivelse af mundtlig information og indhentelse af samtykke

Efter at have udvist interesse modtager potentielle forsøgspersoner det skriftlige materiale (den vedlagte forsøgspersonsinformation og folderen "før du beslutter dig"). Ugen efter indkaldes forsøgspersonerne til samtale på deres lokale aktivitetscenter, hvor projektgruppens medlemmer supplerer den skriftlige information med mundtlig information om baggrunden for forsøget, formålet og forsøgsprotokollen. Herefter vil en forsøgsansvarlig fra projektgruppen i forbindelse med en personlig samtale give mundtlig information om de anvendte metoder samt eventuelle risici og bivirkninger. Forsøgspersonerne gøres opmærksom på, at de kan medbringe en bisidder til dette møde. Dette møde foregår uforstyrret på et til formålet indrettet kontor på forsøgspersonens aktivitetscenter. Det endelige tilsagn og underskrift på samtykkeerklæringen indhentes efter

minimum 2-3 dages betænkningstid og skal senest være indhentet i forbindelse med 2. kontakt til de forsøgsansvarlige, og før de forsøgsrelaterede procedurer indledes. Det understreges, at man når som helst kan trække sig ud af forsøget.

Andersen TR, Schmidt JF, Nielsen JJ, Randers MB, Sundstrup E, Jakobsen MD, Andersen LL, Suetta C, Aagaard P, Bangsbo J, Krustrup P (2014). Effect of football or strength training on functional ability and physical performance in untrained old men. Scand J Med Sci Sports 24 Suppl 1, 76-85.

Barzilay JI, Abraham L, Heckbert SR, Cushman M, Kuller LH, Resnick HE, Tracy RP (2001). The relation of markers of inflammation to the development of glucose disorders in the elderly: the Cardiovascular Health Study. Diabetes 50, 2384-2389.

Bean JF, Kiely DK, Herman S, Leveille SG, Mizer K, Frontera WR, Fielding RA (2002). The relationship between leg power and physical performance in mobility-limited older people. J Am Geriatr Soc 50, 461-467.

Beck BR & Snow CM (2003). Bone health across the lifespan--exercising our options. Exerc Sport Sci Rev 31, 117-122.

Beyer I, Mets T, Bautmans I (2012a). Chronic low-grade inflammation and age-related sarcopenia. Curr Opin Clin Nutr Metab Care 15, 12-22.

Beyer I, Mets T, Bautmans I (2012b). Chronic low-grade inflammation and age-related sarcopenia. Curr Opin Clin Nutr Metab Care 15, 12-22.

Bickel CS, Cross JM, Bamman MM (2011). Exercise dosing to retain resistance training adaptations in young and older adults. Med Sci Sports Exerc 43, 1177-1187.

Brach JS, Simonsick EM, Kritchevsky S, Yaffe K, Newman AB (2004). The association between physical function and lifestyle activity and exercise in the health, aging and body composition study. J Am Geriatr Soc 52, 502-509.

Cornelissen VA & Fagard RH (2005). Effects of endurance training on blood pressure, blood pressure-regulating mechanisms, and cardiovascular risk factors. Hypertension 46, 667-675.

Cuoco A, Callahan DM, Sayers S, Frontera WR, Bean J, Fielding RA (2004). Impact of muscle power and force on gait speed in disabled older men and women. J Gerontol A Biol Sci Med Sci 59, 1200-1206.

Duncan BB, Schmidt MI, Pankow JS, Ballantyne CM, Couper D, Vigo A, Hoogeveen R, Folsom AR, Heiss G (2003). Low-grade systemic inflammation and the development of type 2 diabetes: the atherosclerosis risk in communities study. Diabetes 52, 1799-1805.

Elbe AM, Strahler K, Krustrup P, Wikman J, Stelter R (2010). Experiencing flow in different types of physical activity intervention programs: three randomized studies. Scand J Med Sci Sports 20 Suppl 1, 111-117.

Esmarck B, Andersen JL, Olsen S, Richter EA, Mizuno M, Kjaer M (2001). Timing of postexercise protein intake is important for muscle hypertrophy with resistance training in elderly humans. J Physiol 535, 301-311.

Faulkner JA, Larkin LM, Claflin DR, Brooks SV (2007). Age-related changes in the structure and function of skeletal muscles. Clin Exp Pharmacol Physiol 34, 1091-1096.

Foldvari M, Clark M, Laviolette LC, Bernstein MA, Kaliton D, Castaneda C, Pu CT, Hausdorff JM, Fielding RA, Singh MA (2000). Association of muscle power with functional status in community-dwelling elderly women. J Gerontol A Biol Sci Med Sci 55, M192-M199.

Freeman DJ, Norrie J, Caslake MJ, Gaw A, Ford I, Lowe GD, O'Reilly DS, Packard CJ, Sattar N (2002). C-reactive protein is an independent predictor of risk for the development of diabetes in the West of Scotland Coronary Prevention Study. Diabetes 51, 1596-1600.

Frontera WR, Hughes VA, Fielding RA, Fiatarone MA, Evans WJ, Roubenoff R (2000a). Aging of skeletal muscle: a 12-yr longitudinal study. J Appl Physiol 88, 1321-1326.

Frontera WR, Suh D, Krivickas LS, Hughes VA, Goldstein R, Roubenoff R (2000b). Skeletal muscle fiber quality in older men and women. Am J Physiol Cell Physiol 279, C611-C618.

Greig CA, Botella J, Young A (1993). The quadriceps strength of healthy elderly people remeasured after eight years. Muscle Nerve 16, 6-10.

Han TS, Sattar N, Williams K, Gonzalez-Villalpando C, Lean ME, Haffner SM (2002). Prospective study of C-reactive protein in relation to the development of diabetes and metabolic syndrome in the Mexico City Diabetes Study. Diabetes care 25, 2016-2021.

Helge EW, Aagaard P, Jakobsen MD, Sundstrup E, Randers MB, Karlsson MK, Krustrup P (2010). Recreational football training decreases risk factors for bone fractures in untrained premenopausal women. Scand J Med Sci Sports 20 Suppl 1, 31-39.

Holviala JH, Sallinen JM, Kraemer WJ, Alen MJ, Hakkinen KK (2006). Effects of strength training on muscle strength characteristics, functional capabilities, and balance in middle-aged and older women. J Strength Cond Res 20, 336-344.

Houston DK, Nicklas BJ, Ding J, Harris TB, Tylavsky FA, Newman AB, Lee JS, Sahyoun NR, Visser M, Kritchevsky SB (2008). Dietary protein intake is associated with lean mass change in older, community-dwelling adults: the Health, Aging, and Body Composition (Health ABC) Study. Am J Clin Nutr 87, 150-155.

Jakobsen MD, Sundstrup E, Krustrup P, Aagaard P (2011). The effect of recreational soccer training and running on postural balance in untrained men. Eur J Appl Physiol 111, 521-530.

Janssen I, Shepard DS, Katzmarzyk PT, Roubenoff R (2004). The healthcare costs of sarcopenia in the United States. J Am Geriatr Soc 52, 80-85.

Jespersen J, Pedersen TG, Beyer N (2003). [Sarcopenia and strength training. Age-related changes: effect of strength training]. Ugeskr Laeger 165, 3307-3311.

Johnston AP, De LM, Parise G (2008). Resistance training, sarcopenia, and the mitochondrial theory of aging. Appl Physiol Nutr Metab 33, 191-199.

Kanis JA & Adami S (1994). Bone loss in the elderly. Osteoporos Int 4 Suppl 1, 59-65.

Krustrup P, Aagaard P, Nybo L, Petersen J, Mohr M, Bangsbo J (2010a). Recreational football as a health promoting activity: a topical review. Scand J Med Sci Sports 20 Suppl 1, 1-13.

Krustrup P, Dvorak J, Junge A, Bangsbo J (2010b). Executive summary: the health and fitness benefits of regular participation in small-sided football games. Scand J Med Sci Sports 20 Suppl 1, 132-135.

Krustrup P, Hansen PR, Andersen LJ, Jakobsen MD, Sundstrup E, Randers MB, Christiansen L, Helge EW, Pedersen MT, Sogaard P, Junge A, Dvorak J, Aagaard P, Bangsbo J (2010c). Long-term musculoskeletal and cardiac health effects of recreational football and running for premenopausal women. Scandinavian journal of medicine & science in sports 20 Suppl 1, 58-71.

Krustrup P, Hansen PR, Randers MB, Nybo L, Martone D, Andersen LJ, Bune LT, Junge A, Bangsbo J (2010d). Beneficial effects of recreational football on the cardiovascular risk profile in untrained premenopausal women. Scandinavian journal of medicine & science in sports 20 Suppl 1, 40-49.

Krustrup P, Nielsen JJ, Krustrup BR, Christensen JF, Pedersen H, Randers MB, Aagaard P, Petersen AM, Nybo L, Bangsbo J (2009). Recreational soccer is an effective health-promoting activity for untrained men. British journal of sports medicine 43, 825-831.

Lexell J, Taylor CC, Sjostrom M (1988). What is the cause of the ageing atrophy? Total number, size and proportion of different fiber types studied in whole vastus lateralis muscle from 15- to 83-year-old men. J Neurol Sci 84, 275-294.

Lobo A, Carvalho J, Santos P (2011). Comparison of functional fitness in elderlies with reference values by Rikli and Jones and after one-year of health intervention programs. J Sports Med Phys Fitness 51, 111-120.

Meng SJ & Yu LJ (2010a). Oxidative stress, molecular inflammation and sarcopenia. Int J Mol Sci 11, 1509-1526.

Meng SJ & Yu LJ (2010b). Oxidative stress, molecular inflammation and sarcopenia. Int J Mol Sci 11, 1509-1526.

Moore DR (2014). Keeping older muscle "young" through dietary protein and physical activity. Adv Nutr 5, 599S-607S.

Ottesen L, Jeppesen RS, Krustrup BR (2010). The development of social capital through football and running: studying an intervention program for inactive women. Scand J Med Sci Sports 20 Suppl 1, 118-131.

Pedersen BK & Saltin B (2006). Evidence for prescribing exercise as therapy in chronic disease. Scandinavian journal of medicine & science in sports 16 Suppl 1, 3-63.

Petersen AM & Pedersen BK (2005). The anti-inflammatory effect of exercise. Journal of applied physiology 98, 1154-1162.

Pijnappels M, Bobbert MF, van Dieen JH (2005). Control of support limb muscles in recovery after tripping in young and older subjects. Exp Brain Res 160, 326-333.

Purath J, Buchholz SW, Kark DL (2009). Physical fitness assessment of older adults in the primary care setting. J Am Acad Nurse Pract 21, 101-107.

Rantanen T, Era P, Heikkinen E (1997). Physical activity and the changes in maximal isometric strength in men and women from the age of 75 to 80 years. J Am Geriatr Soc 45, 1439-1445.

Rosenberg IH (1997). Sarcopenia: origins and clinical relevance. J Nutr 127, 990S-991S.

Roubenoff R (2003). Catabolism of aging: is it an inflammatory process? Curr Opin Clin Nutr Metab Care 6, 295-299.

Santos DA, Silva AM, Baptista F, Santos R, Vale S, Mota J, Sardinha LB (2012). Sedentary behavior and physical activity are independently related to functional fitness in older adults. Exp Gerontol 47, 908-912.

Schaap LA, Pluijm SM, Deeg DJ, Visser M (2006). Inflammatory markers and loss of muscle mass (sarcopenia) and strength. Am J Med 119, 526-17.

Skelton DA, Kennedy J, Rutherford OM (2002). Explosive power and asymmetry in leg muscle function in frequent fallers and non-fallers aged over 65. Age Ageing 31, 119-125.

Toto PE, Raina KD, Holm MB, Schlenk EA, Rubinstein EN, Rogers JC (2012). Outcomes of a multicomponent physical activity program for sedentary, community-dwelling older adults. J Aging Phys Act 20, 363-378.

Visser M, Pahor M, Taaffe DR, Goodpaster BH, Simonsick EM, Newman AB, Nevitt M, Harris TB (2002). Relationship of interleukin-6 and tumor necrosis factor-alpha with muscle mass and muscle strength in elderly men and women: the Health ABC Study. J Gerontol A Biol Sci Med Sci 57, M326-M332.

Whipple RH, Wolfson LI, Amerman PM (1987). The relationship of knee and ankle weakness to falls in nursing home residents: an isokinetic study. J Am Geriatr Soc 35, 13-20.

Bjørner JB, Damsgaard MT, Watt T, Bech P, Rasmussen NK, Kristensen TS, Modvig J and Thunedborg K**.** Dansk manual til SF-36. Et spørgeskema om helbredsstatus. *København: Lif Lægemiddelindustriforeningen,* 1997.

Bowling A. The psychometric properties of the Olders People's Quality of Life Questionnaire, compared with the CASP-19 and WHOQOL-OLD. Current Gerontology and Geriatrics Research. 2009: 1-12.

Carron AV, Brawlet LR and Widmeyer NW. The Group Envinronment Questionnaire. Test manual. *Morgantown, WV: Fitness Information Technology*, 2002.

Craig CL, Marshall AL, Sjostrom M, Bauman A, Booth ML, Ainsworth BE, Pratt M, Ekelund U, Yngve A, Sallis JF, Oja P: International Physical Activity Questionnaire: 12-country reliability and validity. Medicine and Science in Sports and Exercise 2003, 35:1381-1395

Kendzierski D, DeCarlo, KJ. Physical Activity Enjoyment Scale: Two validation studies. Journal of Sport & Exercise Psychology. 1991: 13: (1): 50-64.

Ottesen L and Skjerk O. Inaktivitetsundersøgelse. En sammenfatning. *Det Nationale Råd for Folkesundhed og Indenrigs- og Sundhedsministeriet*, 2006.

Pelletier LG, Fortier SF, Vallerand RJ, Tuson KM, Brière NM and Blais MR. Toward a new measure of intrinsic motivation, extrinsic motivation and amotivation in sports: The Sport Motivation Scale (SMS). *Journal of Sport and Exercise Psychology* 17: 35-53, 1995.

Rheinberg F, Vollmeyer R, Engeser S. Die Erfassung des Flow-Erlebens. In: Stiensmeier-Pelster J, Rheinberg F, eds. Diagnostik von Motivation und Selbstkonzept. Göttingen: Hogrefe, 2003: 261–279.

Snaith RP, Zigmond AS. The Hospital Anxiety and Depression Scale. *London: GL assessment,* 1994.
