# Supplementary material for: Effect of small-sided team sport training and protein intake on muscle mass, physical function and markers of health in older untrained adults: A randomized trial
Source: PLoS One. 2017 Oct 10;12(10):e0186202. doi: 10.1371/journal.pone.0186202 (PMC5634648; doi:10.1371/journal.pone.0186202)
Supplement: S3 File — (PDF) [file pone.0186202.s003.pdf]

Study protocol_version 9_20. January 2016

Application to the local ethics committee of Copenhagen

Regionsgården

Kongens Vænge 2

3400 Hillerød

The effect of small-sided ball games with dietary protein on muscle mass, health markers, functional work capacity, and quality of life of senior citizens in municipal activity centers

Study description

Study aim

The overall aim is to examine the effect of small-sided ball games combined with protein intake on muscle and bone mass, changes in general health profile and the functional work capacity in with senior citizens over 65 years in municipal activity centers. In addition, to examine how small-sided ball games affects the inflammatory profile and how this relates to changes in muscle and bone mass, functional work capacity and health markers.

Furthermore, the participants' social and sports backgrounds, their intention to participate, their experiences of participation and their evaluation after the intervention is examined. There will be a specific focus on how team play can help to improve participants' quality of life, self-perceived physical and mental health, and if it can strengthen the cohesion of the group and prevent dropouts in sports participation among older adults.

Hypothesis

The project's main hypotheses is that regular participation in small-sided ball games with protein intake in older adults aged 65 years or older will lead to, (I) favorable changes in body composition, including loss of body fat and visceral fat, and maintained or increased muscle mass, (ii) a more favorable lipid profile shown as a decrease in blood triglycerides and increased HDL / LDL cholesterol ratio, (III), increased bone density, (IV) improved functional work capacity (muscle strength, mobility, time endurance, balance and sit to stand capacity), (V) a reduced level of systemic inflammation. In addition, it is hypothesized that small-sided ball game training will lead to increased (VI) continuation in physical activity, (VII) improvement in self-perceived health and mental health, (VIII) enjoyment of the activity, (IX) group connectedness and (X) higher motivation to participate in the activities.

Introduction

Loss of muscle mass is age-related and accelerates from about 65 years of age (Lexell et al. 1988; Frontera et al. 2000b), which is accompanied by a significant decrease in muscle strength (Rosenberg 1997). With increasing muscle atrophy, the maximum muscular force as well as the ability to develop quick force is reduced (Frontera et al. 2000a; Jespersen et al. 2003), and this is associated with a substantial loss of functional ability, such as loss of balance, to go up the stairs, stand up from a chair and falls (Bean et al. 2002; Cuoco et al. 2004; Foldvari et al. 2000; Holviala et al. 2006; Pijnappels et al. 2005; Skelton et al. 2002; Whipple et al. 1987). Furthermore, loss of muscle mass leads to a substantial decline in the ability to perform activities of daily living as well as an increased risk of falls and fractures, which undermines the independence of the older adults (Faulkner et al. 2007). Loss of bone mass is also age-related and accelerates especially after menopause in both men and women (Kanis & Adami 1994; Beck & Snow 2003), which is shown to be related to the number of bone fractures and falls in older people. Taken together, these factors lead to an increased risk of cardiovascular and musculoskeletal disease that may manifest in an increased incidence of type II diabetes, hypertension, bone loss and impaired postural balance.

Loss of muscle mass and function in older adults is also a socio-economic challenge. Thus, it is estimated that exercise and rehabilitation interventions with a duration of 6 to 8 weeks can reduce a Danish municipality with health expenditures equivalent to 13 million kr. annually (DSI 2011) and in the US alone, the annual health care costs related to loss of muscle mass and function amounts to $ 20 billion (Janssen et al. 2004). In 2004, approximately 41.000 inquiries to the Danish emergency rooms was related to falls in older adults (65+ years), of which about 12.000 led to hospitalization (NIPH 2005). Thus, interventions to reduce impaired motor function will not only be of great personal value for the individual older but also have high economic value for the society.

In older people who exercise regularly, a preventive effect against age-related loss of muscle mass is observed (Bickel et al. 2011), but the precise mechanisms for this is unknown. It is also observed that regular physical activity partly prevents a loss of muscle strength, which may lead to a maintained or improved functional capacity in older adults, and this effect exists despite the presence of other health problems (Greig et al., 1993; Rantanen et al. 1997). The effect has been shown to be greatest in those older adults, who regularly performed exercise at a high intensity compared with everyday activities at lower intensities (Brach et al. 2004). It is unclear how regular physical activity contributes to counteract age-related loss of muscle mass in the elderly. In recent years, however, there has been more focus on the possible role of inflammatory mediators in the development of age-related muscle loss (Beyer et al. 2012a; Roubenoff 2003; Beyer et al. 2012a; Meng & Yu 2010a). Thus, increasing levels of plasma TNF alpha (Tumor Necrosis Factor α), other pro-inflammatory cytokines (IL-6, IL-1) and markers of inflammation (CRP, C-Reactive Protein) has been associated with increasing age (Beyer et al. 2012b; Meng & Yu 2010b) and, in particular, have been shown an association between high serum level of pro-inflammatory cytokines and low muscle strength (Visser et al. 2002). However, it is not yet clear whether the increased TNF-alpha levels observed in older adults is a result of aging, chronic illness or inactivity. By contrast, it seems certain that TNF-alpha has a catabolic effect on skeletal muscle (Schaap et al. 2006) and chronic inflammation has also been associated with metabolic syndrome, type II diabetes and arteriosclerosis (Petersen & Pedersen 2005; He et al. 2002 ; Freeman et al. 2002; Barzilay et al., 2001; Duncan et al. 2003).

A suboptimal protein intake in older adults is associated with increased risk of sarcopenia (Houston et al. 2008). At the same time, studies point out an increased protein intake in older adults, or a specific supplement of essential amino acids, including the branched-chain amino acid leucine, is capable of inducing an anabolic response in muscle protein synthesis after exercise, which is comparable to that obtained in younger people (Moore 2014). Studies among untrained older adults also points out that it is important to consume the protein-rich meal shortly after training, to achieve the greatest effect on lean body mass (Esmarck et al. 2001).

To date, research related to health and physical capacity have mainly been focused on traditional exercise types, like running and cycling (Cornelissen & Fagard 2005; Pedersen & Saltin 2006) and the focus has been largely on resistance training in the treatment of age-related loss of muscle mass (Johnston et al . 2008). Recent studies, however, indicate that exercise forms with an individual touch, such as resistance training and running, do not maintain the individual in the activities compared to social forms of activities such as team play (Krustrup et al. 2010b). In addition, a general loss of quality of life and self-perceived physical and psychological well-being appear to exist with increased age (Bjørner et al. 1997). Therefore, it is necessary to examine whether a social exercise type, such as small-sided ball games, may help to counteract the above described age-related effects throughout life. In this context, it is shown that soccer and small-sided ball games can lead to a number of positive mental (Elbe et al. 2010) and social effects (Ottesen et al. 2010), including greater social cohesion (Carron et al. 2002), greater immersion in the activities (19) and higher motivation to participate in the activities (Pelletier et al. 1995). In a study of inactive Danes, key motivators for participation in physical activity were linked to positive social experiences and previous sports experiences (Ottesen and Skjerk 2006).

Recently, Danish research has shown that soccer can improve the health and physical capacity (Krustrup et al. 2010a; Helge et al. 2010; Krustrup et al. 2010c). It is interesting that a group of older untrained men aged 63-74 years showed the same positive improvements in functional capacity as a resistance training group after 16 weeks of recreational soccer on

small pitches (Andersen et al. 2014). Furthermore, it is shown that small-sided soccer can improve postural balance (Jakobsen et al. 2011). However, the is a lack of information regarding the effects and feasibility of (I) other small-sided ball games than soccer, (II) small-sided ball games in frail or older adults with low physical capacity, including women, and (III) small-sided ball game training on quality of life, including social and mental well-being.

Overall, there is a need to study small-sided ball game training as an activity to promote muscle mass, functional work capacity and quality of life. The study may also contribute with important knowledge about the influence of systemic inflammation on health and physical function of older citizens. This study also differs from previous small-sided ball game studies by being conducted at a real-life platform, allowing synergy between local municipal activity centers and the established free sport associations.

Study design

The study is a randomized exercise trial in municipal activity centers with small-sided ball games as an intervention for 12 months in 80 untrained men and women aged 65+ years. 40 participants will be randomized to a small-sided ball game group and 40 to a control activity group performed as resistance training, which is already offered in the activity centers. The participants are recruited via the Copenhagen Municipality activity centers (elaborated in the recruitment of participants section). The small-sided ball game group exercise 2-3 times a' week of 16 to 24 minutes during the intervention period and training will be performed indoors or outdoors via small-sided ball games, consisting of various customized team games. The training takes place at selected local activity centers in Copenhagen, where the recruited members of the centers already are affiliated, which ensures that the participants are not burdened with additional transport to the site of intervention. Tests are conducted before the intervention period and after 3, 6 and 12 months. Blood samples and DXA scanning will be performed only before and after 12 weeks. Thus, the test at 6 and 12 months will only be non-invasive tests, that is, interviews, questionnaire filling and the determination of functional work capacity.

Participants

80 untrained men and women aged 65+ years will be recruited.

Exclusion criteria:

(I) Participants with serious disease complications, including significant polyneuropathy, cancer, known ischemic heart disease or musculoskeletal disorders that hinder participation in team games. However, well-controlled hypertension, dyslipidemia, and obesity and other age-related chronic symptoms are not contraindicative.

(II) The participants are expected to have a reasonable degree of mobility, i.e., the participants should be able to go without essential use of aids like wheelchair or walker.

(III) The participants must not be in abuse of any kind, including alcohol (maximum 21 drinks per week) and drugs (smoking is not an exclusion criterion).

Methods

A total of 4 test rounds (before the intervention period and after 3, 6 and 12 months) will be conducted. Blood samples and DEXA scanning is carried out only in test round 0 and 3 months. Thus, the test round 6 and 12 months only include the questionnaires, interviews, and measurement of the functional work capacity. The test rounds are described in detail below.

Description of test round 0 and 3 months

The participants participate in two test days, which are separated by at least 48 hours. Participants must not have performed strenuous physical work as well as drinking alcohol the day before the test day, and is not allowed to drink alcohol, tea, coffee, or smoke before the investigations on the test day.

Test day 1:

At the Department of Exercise and Nutrition, University of Copenhagen, University Park, 13, 2nd floor, a health investigation is performed including a resting blood sample and determination of body composition by DEXA scanning. On test day 1, the participants reports in during morning in the fasting state and is after the tests offered a light lunch. The duration of the test day 1 is about 3 hours.

During the blood samples, a venous catheter is placed in the elbow for removal of a total of 50 ml of blood to be able to evaluate the temporal development of cholesterol, triglycerides, blood glucose, insulin, HbA1c, highly sensitive CRP, TNF-alpha, IL-6, IL -1beta and IL-10. Blood samples are only taken during test round 0 and 3 months, which means that a total of 100 ml of blood is taken through the entire project period for a participant.

A full body DEXA scan is performed, which is a scanning that uses weak X-rays, to determine body composition of the different tissue types measured (body fat percentage, muscle mass and bone density). A DEXA scan does not hurt and is not associated with any kind of discomfort.

Test day 2:

In the subject's local municipal activity center, the "Senior fitness test" and questionnaires as well as interviews are conducted. Duration of test day 2 is approximately 3 hours.

The Senior Fitness Test is a simple battery of tests designed to measure functional fitness (i.e. the functional characteristics that are important for functional capacity in the older years). The tests measures the muscular strength (below + upper body), aerobic endurance, flexibility, agility and dynamic balance at all levels of healthy elderly. The test shows the results on a continuous scale, and thus, can demonstrate changes over time in connection with, for example, an exercise intervention. The test battery can be carried out in the "field" and does not require special laboratory facilities. The test battery is used in a number of scientific studies (Toto et al. 2012; Santos et al. 2012; Purath et al. 2009; Lobo et al. 2011) and can be considered a kind of standard for the assessment of older adults physical capacity

outside laboratory settings. Below is a brief description of the selected parts of the senior fitness test, which are expected to be used:

Sit-to stand (strength in the lower body)

The number of times a person repeatedly can sit to stand on a chair within 30 seconds with arms folded against the chest.

Arm curls (strength in the upper body)

The number of arm curls that can be performed within 30 seconds with a dumbbell (≈3,63 kg).

6-minute walk test (aerobic endurance)

Distance which can be covered in 6 minutes on a 46 meter track.

2.45 m up and go test (agility and dynamic balance)

Number of seconds it takes to get up from a sitting position (in the chair), walking 2.45 meters, turn and return to a sitting position.

Lower body flexibility

Sitting on a chair, the participant tries to reach the toes with outstretched legs. Number of centimeters plus or minus between the fingertips and toes are measured. The result is the best of two attempts.

Upper body flexibility

One hand is placed over the shoulder and is moved as far as possible down the back, while the other hand is moved as high as possible up the back from below. The distance in centimeters between the third finger of the two hands is measured. The result is the best of two attempts.

Walking speed grip strength

Hand grip strength is measured in kg with the dominant hand using a handgrip dynamometer (Takei Scientific Instruments Co. Ltd., Tokyo, Japan). Standing with the wrist in a neutral position and the elbow stretched, 3 trials is performed separated by 1 minute between each try. The best of three attempts is registered.

Flamingo test

The subject should stand on one leg over a period of 60 seconds with the aim of measuring postural balance. The number of times the participant loses balance during the 60 seconds is registered.

Tests during the intervention period

Heart rate measurements using Polar heart rate monitors is conducted during selected training sessions.

Validated Danish translations of two standardized questionnaires will be used to measure flow (Flow Short Scale: Rheinberg et al., 2003) and the extent to which participants have enjoyed the activities they participated in (Physical Activity Enjoyment Scale: Kendzierski & DeCarlo, 1991), three times during the intervention period. Participants will be informed in advance as a part of the recruitment process, and the responses are anonymous and confidential.

Information about questionnaires

Two surveys of each group in the intervention will be carried out - a baseline study, which maps the participants' socioeconomic status, level of activity and experience with sports, as well as an evaluative study at the end of the experiment. In addition, validated Danish translation of standardized questionnaires is used to measure self-perceived physical and mental health (SF-12: Bjørner et al., 1997), anxiety and depression levels (Hospital Anxiety and Depression Scale: Snaith & Zigmond, 1994) , quality of life (Older People's Quality of Life Questionnaire: Bowling, 2009), level of daily activity (International Physical activity Questionnaire: Craig et al. 2003), and motivation (Sport motivation Scale: Pelletier et al., 1995), at test round 0, 3 , 6 and 12 months.

Information about interviews

A number of individual interviews and focus group interviews with participants and staff from the activity centers in the project will be conducted. The interviews are performed on a voluntary basis and will be anonymous. Recordings of the interviews will be kept confidential, and any statements from them will be pseudonymized. The purpose of these interviews is to uncover participants' experiences of the activities, their social cohesion and motivation, and the activities' impact on the participants' life situation.

Test round 6 and 12 months

Tests at 6 and 12 months are performed as described above in the *description of the test round 0 and 3 months* with the exception of the test day 1. That is, only test day 2 is performed, thus, the blood samples as well as DEXA scanning are not performed at 6 and 12 months.

The small-sided ball game training

The small-sided ball game training is performed outdoors or indoors in gyms or corresponding areas in or around the activity centers. The small-sided ball game training is offered three times a week, but it is not a requirement that you attend all three times, but at least two times per week is preferred. The small-sided ball game training varies between three different team games: "Hulahopbold" “Floorball”, and "Granny Volley".

1) Floorball, which is played 3 on 3 small areas (e.g. 10x15 meter) with intervals of four minutes separated by a 4-minute recovery period.

2) "Hulahop-ball", where the objective is to throw the ball through a hoop by passing the ball to one of the teammates. The game is played 3 on 3 small areas (e.g. 10x15 meter) with intervals of four minutes separated by a 4-minute recovery period.

3) "Granny volley", a kind of age-adjusted volleyball, wherein a soft ball is passed over a net (or any other obstacle lead), and the ball should be intercepted by an opponent team. The game is played 4 against 4 on small areas (e.g. 10x15 meters) and the participants rotate their playing positions on a regular basis.

All small-sided ball games are dosed carefully (starting with 16 minutes of effective playing the first 4 weeks, 20 minutes the following 4 weeks, and 24 min the remaining 4 weeks). Body contact in the small-sided ball games is not allowed, and any injuries are followed up on before training commences. The small-sided ball games are supervised by the activity center's training staff.

Protein intake

Immediately after, and 3 hours after the training team games, half of the participants ingest a 0.2 L milk based protein beverage containing 20 g of protein. This means that participants will consume 40 grams of protein on training days, and a training frequency of 2-3 times a week will result in an extra protein intake of 80 to 120 g per week. The other half of the participants from the small-sided ball games will ingest a 0.5 L isocaloric placebo drink consisting of juice immediately after exercise and 3 hours after.

Resistance training

In the resistance training group, participants participate in the resistance training provided by the local activity center and the resistance training is offered three times a week. It is not a requirement that you attend all three times a week, but at least 2 times. Three exercises are performed every session. 1) Leg press, 2) shoulder press, and 3) Arm curls. Each exercise is performed 3x8 RM (Maximum reiteration; indicates that the load is adjusted such that a maximum of 8 repetitions can be lifted) and the load is adjusted continuously throughout the program. A recovery of 2-3 minutes between each set is carried out. The resistance training is supervised by the activity center's training staff.

Biobank, handling of biological material and personal data

The purpose of the research biobank is to collect and store biological material. The blood samples are used for a wide range of analysis and there will probably not be any excess biological material left after the planned analysis. Any excess biological material will be destroyed within 6 months after the study period. Participant data are protected under the law of personal data and health. Each volunteer will after study be able to access their own data as well as average data for the whole study group. In the written information for participants, the participants are asked if they want access to their own experimental data no later than 2 months after study has ended. All data will be anonymised.

Statistical considerations and power calculations

Changes in the endpoints before the intervention is determined by using a two-tailed unpaired t-test. Changes between and within groups is determined by a two-way ANOVA and linear regression analysis. When detecting an overall significant interaction (time versus Group) or time-effect, a Student-Newman-Keuls post hoc analysis is used to isolate the group to which the change took place.

The number of subjects is chosen based on the expectation that 75% of the participants will complete the intervention, or at least 30 participants per experimental group, and the

standard deviations of the change in the power calculations are based on observed changes in the research group from similar studies (Krustrup et al., 2009; Krustrup a al. 2010D).

Estimated detection limit for changes in selected main outputs between independent groups (n = 30). ANOVA.

Variable	SD of changes	2 groups
Fat mass (kg)	6.1	4.4
Muscle mass (kg)	2.8	2.1
6-min walking test (m)	110	80

Power is 0.8 and P is 0.05

Side effects, risks and disadvantages for the subjects

DEXA scanning emits weak X-rays, in which the body composition of different tissue types is measured. 0.0006 mSv is emitted by whole body DEXA scan. This is equivalent to a radiation dose that provides an increased cancer risk of 0.001%, which is considered to be a minimal risk. The radiation dose that is imposed to the subjects is smaller than most X-ray examinations and equivalent to 7 days of background radiation. DEXA scans do not hurt and is not associated with any kind of discomfort.

Blood samples are taken from an arm vein via a catheter in the elbow vein. During removal of the catheters, a slight bleeding may lead to discoloration of the skin may occur. To minimize this inconvenience, a compression will be undertaken (light pressure on the site of blood sampling). A total of 100 mL blood is taken (50 ml when tested in round 0 and 50 ml when tested after 3 months) during the entire test period of 12 months. To comparison, 500 ml is taken during a single blood donation. Using catheters are used routinely at the Institute of Sports Science and Nutrition, University of Copenhagen, and has been used without significant complications in more than 30 years.

Risk of performing functional tests in the activity centers is considered minimal. All tests are previously performed in older adults, including Parkinson's patients. Sometimes muscle soreness can occur, corresponding to regular exercise soreness.

Performance of team game activities are considered, as in soccer on small-sided pitches, as a health-promoting activity. By organizing the team games as special customized small-sided games (no body contact, small pitches, few participants on each team, recovery periods etc.), it is well-known that the risk of injuries is minimized considerably compared to e.g. traditional football matches (Krustrup et al. 2010a). The small-sided ball games are supervised by the activity center's training professionals. It is well known that untrained individuals can experience muscle soreness after the start of a training program. This is a natural process, and typically decreases after 2-4 weeks. Muscle strain (including posterior and anterior thigh), overload of tendons and injuries (e.g. in ankles and knees) may occur in rare cases during team play training on small areas. Lessons from previous team play studies show, however, that small-sided recreational soccer (which is considered a comparable activity to small-sided ball game training) is a relatively safe form of exercise for untrained individuals, including the elderly, and only few injuries are typically reported.

Performing resistance training is considered to have minimal risk, and is considered a health-promoting and physical function promoting activity of older individuals, why resistance training also is a part of the exercise recommendations from the Danish authorities of national health. Resistance training takes place under controlled conditions using machines, reducing the risk of injuries to a minimal. Strength training is supervised by the activity center's training professionals.

The study initiators and the staff behind the study

The study origins from the department of Nutrition, Exercise and Sports as well as Copenhagen Center for team sports and Health, University of Copenhagen. Initiator of the study is Professor Jens Bangsbo and the municipality of Copenhagen in collaboration with Danish Gymnastics and Sports Associations (DGI). The scientific study staff consists of

Professor Jens Bangsbo, Professor Gertrud Pfister, Associate Professor Mogens Theisen Pedersen, Ph.D. stud. Jacob Vorup, AC Pia Sandfeld Melcher, research assistant Johan Michael Wikman and research assistant Anne Nistrup, all from the Department of Nutrition, Exercise and Sports, University of Copenhagen. The project is conducted in cooperation with the municipality of Copenhagen and DGI.

Funding

The study is fully funded, of which two thirds of the study is funded by the municipality of Copenhagen, while the last third is financed by the Copenhagen Center for team play and Health, University of Copenhagen. Study funds are paid to a research account at the department of Nutrition, Exercise and Sports, University of Copenhagen and used partly for the purchase of equipment for blood analysis, and salary for a research assistant. There are also allocated for overhead. No one in the project have conflicts of interest.

Participants and study fees

There will be no financial fees for trial participants. However, the project covers the necessary equipment and materials for performing the team game training (e.g. balls, floorball sticks and goals etc.). Also, any transportation expenses to the department of Nutrition, Exercise and Sports, University of Copenhagen will be covered.

Dissemination of test results

The study results will be widely disseminated and can already from spring 2016 be continuously incorporated into the political debate through a series of lectures, conferences and other events in municipalities and sport associations. The results should be communicated both externally, e.g. via press conferences or other media based publication, as well as internally in the Danish municipalities and sports associations, e.g. through DGI. Results of the study is also expected to be published in a number of popular articles, e.g. in

DGI's magazine UDSPIL and other relevant journals. It is also planned that the results should be public through a series of health conferences where future municipal-based health promotion is up for debate. Both the positive, negative and inconclusive results will be published.

Insurance

All participants in the study are covered by the patient injury insurance in H:S.

Ethical considerations and respect for the subjects' physical and mental integrity

The experiment will be approved by the ethical committee. Team play activities and resistance training are considered to be beneficial for health and physical fitness. Blood samples (maximum 50 ml blood, respectively, in each test round at 0 and 3 months, corresponding to approximately 1% of total blood volume) is used to investigating the hypothesis of the study and are not considered an ethical problem for a group of older individuals 65+ years.

The experiment is performed in accordance with the Declaration of Helsinki and the experiment is reported to the Data Protection Agency. Subject data is protected under the law of personal data and health. Any excess biological material will be destroyed.

Participant information and obtaining consent

After having shown interest the study, potential subjects will receive the written material. The following week, the volunteers are invited to an interview at their local activity center where the project team staff will provide verbal information about the background to the study, the purpose and the study protocol. Then, an investigator from the project will provide oral information at a personal level regarding the methods applied and the possible risks and side

effects of the study. The subjects are reminded that they can bring a companion to this meeting. The final commitments and signature on the consent form is obtained after a minimum of 2-3 days of reflection and is collected during the second contact with the investigator, and before the trial is initiated. It is emphasized that a subject can withdraw from the trial at any time.

References

Andersen TR, Schmidt JF, Nielsen JJ, Randers MB, Sundstrup E, Jakobsen MD, Andersen LL, Suetta C, Aagaard P, Bangsbo J, Krustrup P (2014). Effect of football or strength training on functional ability and physical performance in untrained old men. Scand J Med Sci Sports 24 Suppl 1, 76-85.

Barzilay JI, Abraham L, Heckbert SR, Cushman M, Kuller LH, Resnick HE, Tracy RP (2001). The relation of markers of inflammation to the development of glucose disorders in the elderly: the Cardiovascular Health Study. Diabetes 50, 2384-2389.

Bean JF, Kiely DK, Herman S, Leveille SG, Mizer K, Frontera WR, Fielding RA (2002). The relationship between leg power and physical performance in mobility-limited older people. J Am Geriatr Soc 50, 461-467.

Beck BR & Snow CM (2003). Bone health across the lifespan--exercising our options. Exerc Sport Sci Rev 31, 117-122.

Beyer I, Mets T, Bautmans I (2012a). Chronic low-grade inflammation and age-related sarcopenia. Curr Opin Clin Nutr Metab Care 15, 12-22.

Beyer I, Mets T, Bautmans I (2012b). Chronic low-grade inflammation and age-related sarcopenia. Curr Opin Clin Nutr Metab Care 15, 12-22.

Bickel CS, Cross JM, Bamman MM (2011). Exercise dosing to retain resistance training adaptations in young and older adults. Med Sci Sports Exerc 43, 1177-1187.

Brach JS, Simonsick EM, Kritchevsky S, Yaffe K, Newman AB (2004). The association between physical function and lifestyle activity and exercise in the health, aging and body composition study. J Am Geriatr Soc 52, 502-509.

Cornelissen VA & Fagard RH (2005). Effects of endurance training on blood pressure, blood pressure-regulating mechanisms, and cardiovascular risk factors. Hypertension 46, 667-675.

Cuoco A, Callahan DM, Sayers S, Frontera WR, Bean J, Fielding RA (2004). Impact of muscle power and force on gait speed in disabled older men and women. J Gerontol A Biol Sci Med Sci 59, 1200-1206.

Duncan BB, Schmidt MI, Pankow JS, Ballantyne CM, Couper D, Vigo A, Hoogeveen R, Folsom AR, Heiss G (2003). Low-grade systemic inflammation and the development of type 2 diabetes: the atherosclerosis risk in communities study. Diabetes 52, 1799-1805.

Elbe AM, Strahler K, Krustrup P, Wikman J, Stelter R (2010). Experiencing flow in different types of physical activity intervention programs: three randomized studies. Scand J Med Sci Sports 20 Suppl 1, 111-117.

Esmarck B, Andersen JL, Olsen S, Richter EA, Mizuno M, Kjaer M (2001). Timing of postexercise protein intake is important for muscle hypertrophy with resistance training in elderly humans. J Physiol 535, 301-311.

Faulkner JA, Larkin LM, Claflin DR, Brooks SV (2007). Age-related changes in the structure and function of skeletal muscles. Clin Exp Pharmacol Physiol 34, 1091-1096.

Foldvari M, Clark M, Laviolette LC, Bernstein MA, Kaliton D, Castaneda C, Pu CT, Hausdorff JM, Fielding RA, Singh MA (2000). Association of muscle power with functional status in community-dwelling elderly women. J Gerontol A Biol Sci Med Sci 55, M192-M199.

Freeman DJ, Norrie J, Caslake MJ, Gaw A, Ford I, Lowe GD, O'Reilly DS, Packard CJ, Sattar N (2002). C-reactive protein is an independent predictor of risk for the development of diabetes in the West of Scotland Coronary Prevention Study. Diabetes 51, 1596-1600.

Frontera WR, Hughes VA, Fielding RA, Fiatarone MA, Evans WJ, Roubenoff R (2000a). Aging of skeletal muscle: a 12-yr longitudinal study. J Appl Physiol 88, 1321-1326.

Frontera WR, Suh D, Krivickas LS, Hughes VA, Goldstein R, Roubenoff R (2000b). Skeletal muscle fiber quality in older men and women. Am J Physiol Cell Physiol 279, C611-C618.

Greig CA, Botella J, Young A (1993). The quadriceps strength of healthy elderly people remeasured after eight years. Muscle Nerve 16, 6-10.

Han TS, Sattar N, Williams K, Gonzalez-Villalpando C, Lean ME, Haffner SM (2002). Prospective study of C-reactive protein in relation to the development of diabetes and metabolic syndrome in the Mexico City Diabetes Study. Diabetes care 25, 2016-2021.

Helge EW, Aagaard P, Jakobsen MD, Sundstrup E, Randers MB, Karlsson MK, Krustrup P (2010). Recreational football training decreases risk factors for bone fractures in untrained premenopausal women. Scand J Med Sci Sports 20 Suppl 1, 31-39.

Holviala JH, Sallinen JM, Kraemer WJ, Alen MJ, Hakkinen KK (2006). Effects of strength training on muscle strength characteristics, functional capabilities, and balance in middle-aged and older women. J Strength Cond Res 20, 336-344.

Houston DK, Nicklas BJ, Ding J, Harris TB, Tylavsky FA, Newman AB, Lee JS, Sahyoun NR, Visser M, Kritchevsky SB (2008). Dietary protein intake is associated with lean mass change in

older, community-dwelling adults: the Health, Aging, and Body Composition (Health ABC) Study. Am J Clin Nutr 87, 150-155.

Jakobsen MD, Sundstrup E, Krustrup P, Aagaard P (2011). The effect of recreational soccer training and running on postural balance in untrained men. Eur J Appl Physiol 111, 521-530.

Janssen I, Shepard DS, Katzmarzyk PT, Roubenoff R (2004). The healthcare costs of sarcopenia in the United States. J Am Geriatr Soc 52, 80-85.

Jespersen J, Pedersen TG, Beyer N (2003). [Sarcopenia and strength training. Age-related changes: effect of strength training]. Ugeskr Laeger 165, 3307-3311.

Johnston AP, De LM, Parise G (2008). Resistance training, sarcopenia, and the mitochondrial theory of aging. Appl Physiol Nutr Metab 33, 191-199.

Kanis JA & Adami S (1994). Bone loss in the elderly. Osteoporos Int 4 Suppl 1, 59-65.

Krustrup P, Aagaard P, Nybo L, Petersen J, Mohr M, Bangsbo J (2010a). Recreational football as a health promoting activity: a topical review. Scand J Med Sci Sports 20 Suppl 1, 1-13.

Krustrup P, Dvorak J, Junge A, Bangsbo J (2010b). Executive summary: the health and fitness benefits of regular participation in small-sided football games. Scand J Med Sci Sports 20 Suppl 1, 132-135.

Krustrup P, Hansen PR, Andersen LJ, Jakobsen MD, Sundstrup E, Randers MB, Christiansen L, Helge EW, Pedersen MT, Sogaard P, Junge A, Dvorak J, Aagaard P, Bangsbo J (2010c). Long-term musculoskeletal and cardiac health effects of recreational football and running for premenopausal women. Scandinavian journal of medicine & science in sports 20 Suppl 1, 58-71.

Krustrup P, Hansen PR, Randers MB, Nybo L, Martone D, Andersen LJ, Bune LT, Junge A, Bangsbo J (2010d). Beneficial effects of recreational football on the cardiovascular risk profile in untrained premenopausal women. Scandinavian journal of medicine & science in sports 20 Suppl 1, 40-49.

Krustrup P, Nielsen JJ, Krustrup BR, Christensen JF, Pedersen H, Randers MB, Aagaard P, Petersen AM, Nybo L, Bangsbo J (2009). Recreational soccer is an effective health-promoting activity for untrained men. British journal of sports medicine 43, 825-831.

Lexell J, Taylor CC, Sjostrom M (1988). What is the cause of the ageing atrophy? Total number, size and proportion of different fiber types studied in whole vastus lateralis muscle from 15- to 83-year-old men. J Neurol Sci 84, 275-294.

Lobo A, Carvalho J, Santos P (2011). Comparison of functional fitness in elderlies with reference values by Rikli and Jones and after one-year of health intervention programs. J Sports Med Phys Fitness 51, 111-120.

Meng SJ & Yu LJ (2010a). Oxidative stress, molecular inflammation and sarcopenia. Int J Mol Sci 11, 1509-1526.

Meng SJ & Yu LJ (2010b). Oxidative stress, molecular inflammation and sarcopenia. Int J Mol Sci 11, 1509-1526.

Moore DR (2014). Keeping older muscle "young" through dietary protein and physical activity. Adv Nutr 5, 599S-607S.

Ottesen L, Jeppesen RS, Krustrup BR (2010). The development of social capital through football and running: studying an intervention program for inactive women. Scand J Med Sci Sports 20 Suppl 1, 118-131.

Pedersen BK & Saltin B (2006). Evidence for prescribing exercise as therapy in chronic disease. Scandinavian journal of medicine & science in sports 16 Suppl 1, 3-63.

Petersen AM & Pedersen BK (2005). The anti-inflammatory effect of exercise. Journal of applied physiology 98, 1154-1162.

Pijnappels M, Bobbert MF, van Dieen JH (2005). Control of support limb muscles in recovery after tripping in young and older subjects. Exp Brain Res 160, 326-333.

Purath J, Buchholz SW, Kark DL (2009). Physical fitness assessment of older adults in the primary care setting. J Am Acad Nurse Pract 21, 101-107.

Rantanen T, Era P, Heikkinen E (1997). Physical activity and the changes in maximal isometric strength in men and women from the age of 75 to 80 years. J Am Geriatr Soc 45, 1439-1445.

Rosenberg IH (1997). Sarcopenia: origins and clinical relevance. J Nutr 127, 990S-991S.

Roubenoff R (2003). Catabolism of aging: is it an inflammatory process? Curr Opin Clin Nutr Metab Care 6, 295-299.

Santos DA, Silva AM, Baptista F, Santos R, Vale S, Mota J, Sardinha LB (2012). Sedentary behavior and physical activity are independently related to functional fitness in older adults. Exp Gerontol 47, 908-912.

Schaap LA, Pluijm SM, Deeg DJ, Visser M (2006). Inflammatory markers and loss of muscle mass (sarcopenia) and strength. Am J Med 119, 526-17.

Skelton DA, Kennedy J, Rutherford OM (2002). Explosive power and asymmetry in leg muscle function in frequent fallers and non-fallers aged over 65. Age Ageing 31, 119-125.

Toto PE, Raina KD, Holm MB, Schlenk EA, Rubinstein EN, Rogers JC (2012). Outcomes of a multicomponent physical activity program for sedentary, community-dwelling older adults. J Aging Phys Act 20, 363-378.

Visser M, Pahor M, Taaffe DR, Goodpaster BH, Simonsick EM, Newman AB, Nevitt M, Harris TB (2002). Relationship of interleukin-6 and tumor necrosis factor-alpha with muscle mass and muscle strength in elderly men and women: the Health ABC Study. J Gerontol A Biol Sci Med Sci 57, M326-M332.

Whipple RH, Wolfson LI, Amerman PM (1987). The relationship of knee and ankle weakness to falls in nursing home residents: an isokinetic study. J Am Geriatr Soc 35, 13-20.

Bjørner JB, Damsgaard MT, Watt T, Bech P, Rasmussen NK, Kristensen TS, Modvig J and Thunedborg K**.** Dansk manual til SF-36. Et spørgeskema om helbredsstatus. *København: Lif Lægemiddelindustriforeningen,* 1997.

Bowling A. The psychometric properties of the Olders People's Quality of Life Questionnaire, compared with the CASP-19 and WHOQOL-OLD. Current Gerontology and Geriatrics Research. 2009: 1-12.

Carron AV, Brawlet LR and Widmeyer NW**.** The Group Envinronment Questionnaire. Test manual. *Morgantown, WV: Fitness Information Technology*, 2002.

Craig CL, Marshall AL, Sjostrom M, Bauman A, Booth ML, Ainsworth BE, Pratt M, Ekelund U, Yngve A, Sallis JF, Oja P: International Physical Activity Questionnaire: 12-country reliability and validity. Medicine and Science in Sports and Exercise 2003, 35:1381-1395

Kendzierski D, DeCarlo, KJ. Physical Activity Enjoyment Scale: Two validation studies. Journal of Sport & Exercise Psychology. 1991: 13: (1): 50-64.

Ottesen L and Skjerk O. Inaktivitetsundersøgelse. En sammenfatning. *Det Nationale Råd for Folkesundhed og Indenrigs- og Sundhedsministeriet*, 2006.

Pelletier LG, Fortier SF, Vallerand RJ, Tuson KM, Brière NM and Blais MR. Toward a new measure of intrinsic motivation, extrinsic motivation and amotivation in sports: The Sport Motivation Scale (SMS). *Journal of Sport and Exercise Psychology* 17: 35-53, 1995.

Rheinberg F, Vollmeyer R, Engeser S. Die Erfassung des Flow-Erlebens. In: Stiensmeier-Pelster J, Rheinberg F, eds. Diagnostik von Motivation und Selbstkonzept. Göttingen: Hogrefe, 2003: 261–279.

Snaith RP, Zigmond AS. The Hospital Anxiety and Depression Scale. *London: GL assessment,* 1994.
